# Bioengineered hollow nanoflowers to synergistically modulate inflammation, angiogenesis and osteogenesis for enhancing repair of bone defects

**DOI:** 10.1186/s12951-025-03891-0

**Published:** 2025-12-05

**Authors:** Hanyu Sun, Xiaoyu Wang, Pugeng Li, Xinna Wang, Zhengchuan Zhang, Xiaoqiong Huang, Chaoran Fu, Qingci Kong, Lijian Jin, Hai Ming Wong, Feilong Deng, Xuan Li, Xiaolin Yu

**Affiliations:** 1 Hospital of Stomatology, Guanghua School of Stomatology, Sun Yat-sen University, Guangzhou, P. R. China; 2 Guangdong Provincial Key Laboratory of Stomatology, Guangzhou, P. R. China; 3 Faculty of Dentistry, The University of Hong Kong, Hong Kong, Hong Kong SAR P. R. China; 4 Department of Mechanical Engineering, The University of Hong Kong, Hong Kong, Hong Kong SAR P. R. China

**Keywords:** Nanoflowers, ZIF-8, Gallic acid, Anti-inflammation, Angiogenesis, Osteogenesis, Bone regeneration

## Abstract

**Background:**

Inadequate control of inflammation and insufficient vascularization remain major challenges in repair of bone defects. Here, we developed a multifunctional nanoflower, Au NPs@ZIF-8/Ga, by loading gallic acid (Ga) into a nanoflower-like structure consisting of gold nanoparticles (Au NPs) core and zeolitic imidazolate framework-8 (ZIF-8) shell, to synergistically exert anti-inflammatory, pro-angiogenic, and osteogenic effects.

**Results:**

The hollow architectures of the synthesized Au NPs@ZIF-8/Ga nanoflowers were characterized by transmission electron microscopy (TEM), energy-dispersive spectroscopy (EDS), X-ray diffraction (XRD), Fourier-transform infrared spectroscopy (FTIR), and nitrogen adsorption–desorption analysis. In vitro studies demonstrated that Au NPs@ZIF-8/Ga reduced secretion of pro-inflammatory cytokines in macrophages via suppressing NF-κB pathway activation, while concurrently promoted endothelial cell migration, and tube formation. Yet, Au NPs@ZIF-8/Ga enhanced osteogenic differentiation of MC3T3-E1 cells, as evidenced by the upregulated expression of bone formation related genes runt-related transcription factor 2 (RUNX2) and osteocalcin (OCN), as well as increased alkaline phosphatase (ALP) activity and bone matrix mineralization. In vivo studies showed that Au NPs@ZIF-8/Ga promoted early resolution of inflammation, neovascularization and robust new bone formation in a rat model with critical-sized calvarial defects, as confirmed by Micro-computed tomography (micro-CT) and histological analyses.

**Conclusion:**

Collectively, this work presents a versatile nanoplatform for reducing inflammation in early stage while subsequently promoting angiogenesis and osteogenesis, thereby offering a promising therapeutic strategy for bone regeneration under inflammatory conditions.

**Graphical abstract:**

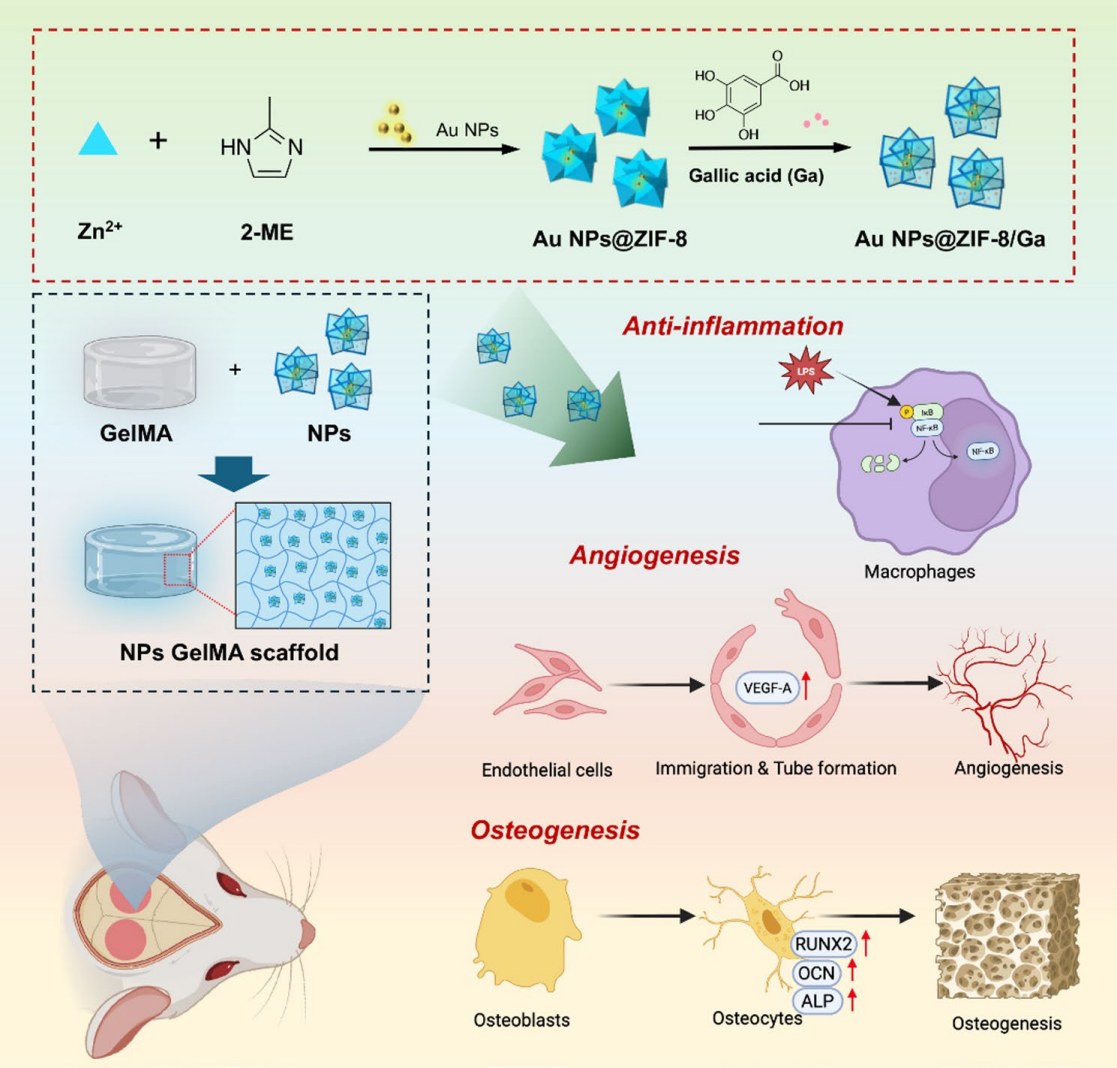

**Supplementary Information:**

The online version contains supplementary material available at 10.1186/s12951-025-03891-0.

## Background

Bone defects constitute a major global clinical challenge, affecting countless individuals annually due to trauma, tumors, inflammatory diseases, or congenital abnormalities, leading to impaired function and reduced quality of life [1]. Bone augmentation surgery, including autologous grafting, bone substitutes, and engineered scaffolds, is widely used to restore bone volume and regeneration [2–4]. Among these, autologous bone grafting remains the gold standard due to its inherent osteoconductive, osteoinductive, and osteogenic properties. However, its clinical application is limited by donor site morbidity, restricted availability, and the need for additional surgical procedures [5]. While synthetic bone substitutes and biomaterial-based scaffolds offer good biocompatibility and structural support, many currently available materials lack key biological functionalities necessary for optimal regeneration [6]. In particular, the limited capacity to modulate inflammation, promote angiogenesis, and support osteogenesis often compromises their overall therapeutic efficacy [7]. These shortcomings are especially critical during the early postoperative phase, as insufficient vascularization, persistent inflammation, and poor integration with host tissue significantly impede bone healing [8, 9].

Following bone grafting surgery, bone regeneration progresses through a precisely timed cascade of biological events involving coordinated cellular interactions across distinct phases. During the early postoperative period prior to complete soft tissue closure, although the initial brief and mild inflammatory stage is crucial for the normal healing of bones, excessive acute inflammation and persistent chronic inflammation would have a negative impact on the bone tissue regeneration process, and may even lead to delayed healing [10]. Moreover, artificial biomaterials may even cause a foreign body reaction after implantation, and the degradation products of the implanted materials may also lead to inflammation [11]. The unregulated inflammatory responses can lead to the failure of the bone repair process, and even cause secondary infections and complications [10]. Therefore, the anti-inflammatory property of bone graft materials is of great significance during the initial implantation and integration stage.

Meanwhile, endothelial cell migration initiates vascularization at the bone defect site, and the neovascularization is an essential reparative mechanism by delivering oxygen and nutrients while sustaining osteoblast activity [12]. Endothelial proliferation and directional migration establish functional vascular networks and create a microenvironment conducive to the subsequent osteogenic process [13]. Importantly, osteoprogenitor cells are recruited to the defect site after injury, where they proliferate and progressively differentiate into pre-osteoblasts and mature osteoblasts. This stepwise process involves matrix secretion, subsequent mineralization, and maturation, ultimately culminating in the formation of new bone tissue [14]. As such, optimal bone augmentation materials should possess tripartite functionality, with concurrent anti-inflammatory, pro-angiogenic, and osteogenic properties to dynamically support the sequential regenerative cascade [15].

Zeolitic imidazolate framework-8 (ZIF-8), a kind of metal-organic framework (MOF), has emerged as a promising material for bone tissue engineering due to its unique physicochemical properties and multifunctional adaptability [16, 17]. Its ability of releasing zinc ions (Zn²⁺) not only activates signaling pathways to stimulate osteoblast proliferation and differentiation [18]. but also promotes endothelial cell growth and migration, thereby supporting angiogenesis and nutrient supply during bone regeneration [19]. Additionally, the unique porous structure of ZIF-8 imparts a high specific surface area, enabling efficient loading of functional molecules. Loading bioactive agents, such as Thymosin β10 or anti-inflammatory agent dexamethasone, into ZIF-8, can further enhance the therapeutic potential of ZIF-8 through the controlled release of the loaded bioactive molecules [20, 21].

Gold nanoparticles (Au NPs) have emerged as a versatile platform in regenerative medicine owing to their bioactive properties and multifunctional capabilities [22, 23]. Au NPs enhance osteogenesis through modulation of key signaling pathways (e.g., ERK/MAPK), promoting osteoblast proliferation, differentiation, and mineralization while concurrently suppressing adipogenic processes [24]. Beyond bone repair, Au NPs serve as antibacterial agents, imaging enhancers, and photothermal converters owing to their strong surface plasmon resonance and facile functionalization [25, 26]. Moreover, the incorporation of Au NPs can alter the structural and functional properties of composite systems by modulating material morphology, improving interfacial interactions, and enhancing overall stability [27], making them valuable components in advanced biomaterial design.

Gallic acid (Ga, 3,4,5-trihydroxybenzoic acid), a natural polyphenolic compound, has gained increasing attention in bone regeneration owing to its multifaceted therapeutic potentials [28]. Ga exhibits potent anti-inflammatory and antioxidant properties and specifically modulates macrophage polarization toward the anti-inflammatory M2 phenotype [29]. Notably, Ga demonstrates osteogenic rescue effects by upregulating alkaline phosphatase (ALP) activity and collagen synthesis in cadmium-impaired osteogenesis models [30]. Furthermore, Ga suppresses osteoclastogenesis and attenuates ovariectomy-induced bone resorption, highlighting its dual regulatory capacity in bone remodeling processes [31]. Despite these advantages, the poor bioavailability of Ga hampers its clinical use. Nanoparticle-based delivery systems offer a promising approach to enhance the therapeutic efficacy of active ingredient in bone regeneration [32].

Although numerous bone substitutes and scaffolds have been developed for repairing alveolar bone defect, existing materials often fail to simultaneously exert anti-inflammatory, angiogenic, and osteogenic effects. Filling this critical gap remains a formidable challenge. The present study seeks to explore innovative strategies to overcome these limitations by designing a nanoflower, in which Ga was loaded in Au NP-embedded ZIF-8, to promote bone repair from different dimensions. First, the anti-inflammatory properties of Ga can effectively mitigate post-surgery induced acute inflammatory responses. Second, the pro-angiogenic effects of ZIF-8 enables vascular network formation during early-stage of bone healing, thus accelerating bone regeneration. Finally, the degradation of such a nanoflower can sustainably release Ga, ZIF-8, and Au NPs to achieve long-term bone repair.

## Experimental section

### Synthesis of Au NPs, ZIF-8, Au NPs@ZIF-8, and Au NPs@ZIF-8/Ga

Au NPs were prepared following a reported citrate reduction method [33, 34]. Trisodium citrate solution (0.1% w/v, 2 mL) was added dropwise to boiling HAuCl_4_ solution (0.001% w/v, 150 mL) until the solution color changed from yellow to wine-red. Polyvinylpyrrolidone (PVP, 0.5 g, M_W_ ~55,000) was then added, and the mixture was stirred at 400 rpm for 24 h. PVP-coated Au NPs were collected by centrifugation (16,000 ×g, 30 min) and washed thrice with methanol.

ZIF-8 particles were synthesized as described previously [35]. Briefly, 1.53 g of Zn(NO_3_)_2_·6H_2_O and 3.62 g of 2-methylimidazole were separately dissolved in methanol. The two solutions were mixed and stirred for 1 h at room temperature. After centrifugation at 10,000 rpm for 20 min, the supernatant was discarded to collect the white precipitate. The precipitate was washed three times with methanol and dried in a vacuum freeze-drier for 24 h.

Au NPs@ZIF-8 was synthesized by mixing PVP-coated Au NPs with Zn(NO_3_)_2_·6H_2_O solution (25 mM, 50 mL). A 2-methylimidazole solution (25 mM, 50 mL) was added dropwise under agitation. The mixture was stirred for 24 h, and particles were collected by centrifugation (8,000 ×g, 30 min).

Au NPs@ZIF-8/Ga was obtained by dispersing 50 mg Au NPs@ZIF-8 in gallic acid (Ga) methanol solution (3 mg/mL, 20 mL). After 24 h stirring, particles were centrifuged (10,000 ×g, 20 min) and freeze-dried for 24 h.

### Characterization of Au NPs@ZIF-8/Ga

The morphology and microstructure of Au NPs, ZIF-8, Au NPs@ZIF-8, and Au NPs@ZIF-8/Ga were examined using Transmission electron microscopy (TEM, Thermo Scientific Talos F200X, USA). Elemental composition was analyzed via Energy-dispersive X-ray spectroscopy (EDS) mapping. Crystallographic features were characterized by X-ray diffraction (XRD, SmartLab SE, Rigaku, Japan), and chemical bonding profiles were assessed using Fourier-transform infrared (FTIR, Nicolet iS50, Thermo Fisher Scientific, USA). Nitrogen adsorption–desorption isotherms were obtained using a surface area and porosity analyzer (ASAP 2460, Micromeritics, USA) to determine Brunauer–Emmett–Teller (BET) specific surface area and pore size distribution of the nanoparticles. The detailed experimental procedures for determining the loading efficiency and release behavior of Ga from Au NPs@ZIF-8/Ga, together with the Zn²⁺ release measurements, are described in the Supplementary Information.

### Biocompatibility assessment of Au NPs@ZIF-8/Ga

Detailed cell culture conditions of MC3T3-E1, human umbilical vein endothelial cells (HUVECs), and RAW 264.7 cells are described in the Supplementary information.

The biocompatibility of Au NPs@ZIF-8/Ga was assessed using MC3T3-E1 osteoblasts, HUVECs, and RAW 264.7 macrophages. Cell culture conditions are provided in the Supplementary Information. Cells were seeded in 96-well plates at 5 × 10³ cells/well and treated with different nanoparticles (ZIF-8, Au NPs@ZIF-8, or Au NPs@ZIF-8/Ga) at 25, 50, 100, and 200 µg/mL for 24 h and 48 h. Equivalent concentrations of free Ga (8, 16, 32, and 64 µg/mL) were calculated based on the Au NPs@ZIF-8/Ga loading efficiency (32.04%). Cell viability was quantified using a CCK-8 assay (CK04, Dojindo, Japan), with absorbance measured at 450 nm using a microplate reader (Epoch 2, BioTek, USA). Controls received culture medium without particles or Ga.

Hemocompatibility was evaluated using fresh whole blood collected from healthy Sprague–Dawley (SD) rats by tail vein sampling, which were obtained in compliance with institutional ethical guidelines and approved by the Ethics Committee for Animal Experiments of Sun Yat-Sen University (Approval No. SYSU-IACUC-2024-001838). After centrifugation at 3,000 ×g for 10 min, red blood cells (RBCs) were washed three times with PBS to prepare a 2% (v/v) suspension. Nanoparticles (25 µg/mL) were incubated with the RBC suspension at 37 °C for 3 h. Absorbance of the supernatant was measured at 541 nm to calculate hemolysis rates. Distilled water and PBS served as positive and negative controls, respectively.

For the fluorescent-based viability assay, cells (2 × 10⁴) were seeded into glass-bottom dishes and treated with nanoparticles (25 µg/mL) or Ga (8 µg/mL). After 24 h, cells were stained with 2 µM Calcein-AM (Solarbio, China) and 4 µM propidium iodide (PI, Solarbio, China) for 30 min at 37 °C, washed with PBS, and imaged using a confocal laser scanning microscope (Olympus FV3000, Japan). Green fluorescence indicated viable cells, and red fluorescence indicated dead ones. Moreover, to assess long-term biosafety at this concentration, we performed a 7-day CCK-8 assay. RAW 264.7, MC3T3-E1, and HUVECs were exposed to nanoparticles (25 µg/mL) or gallic acid (Ga, 8 µg/mL) for 7 days, and cell viability was then measured using the aforementioned CCK-8 protocol. This concentration was used for all subsequent in vitro experiments due to its minimal cytotoxicity.

### *In vitro* anti-inflammatory assessment

An in vitro inflammation model was established using RAW 264.7 macrophages stimulated with lipopolysaccharide (LPS, 1 µg/mL, from *Escherichia coli O55:B5*, Solarbio, China) to mimic acute inflammatory conditions following bone augmentation. In general, cells were pretreated with ZIF-8, Au NPs@ZIF-8, free gallic acid (Ga), or Au NPs@ZIF-8/Ga for 24 h prior to LPS exposure. Untreated cells served as the negative control, while LPS-only treated cells served as the positive control. RAW 264.7 cells were seeded in 6-well plates at a density of 2 × 10⁵ cells/well and assigned to six groups: (1) negative control, (2) positive control (LPS only, 1 µg/mL), (3) ZIF-8 (25 µg/mL) + LPS, (4) Au NPs@ZIF-8 (25 µg/mL) + LPS, (5) Ga (8 µg/mL) + LPS, and (6) Au NPs@ZIF-8/Ga (25 µg/mL, equivalent to 8 µg/mL Ga) + LPS. As described aforementioned, following pretreatment of nanoparticles or Ga for 24 h, cells in groups 2 through 6 were stimulated with LPS for the indicated durations.

For studying the activation of nuclear factor kappa B (NF-κB) pathway, nuclear and cytoplasmic protein fractions were extracted at 30 min post-LPS stimulation, a timepoint commonly used to capture peak NF-κB activation as reported in previous studies [36, 37]. Western blotting was performed to evaluate phosphorylation of IKKα/β (Ser^176/180^), total IKKα/β, and IκBα degradation in cytoplasmic extracts (normalized to β-actin). Nuclear translocation of p65 was examined via both Western blotting (normalized to Histone H3) and immunofluorescence staining using anti-p65 antibody. Detailed protocols for protein extraction, Western blot, and immunofluorescence staining are provided in the Supplementary information.

Regarding the expression of the pro-inflammatory markers, at 24 h post-LPS stimulation, the expression of inducible nitric oxide synthase (iNOS) and tumor necrosis factor-α (TNF-α) was analyzed using immunofluorescence staining. In parallel, Western blot analysis was used to quantify protein levels of iNOS and cyclooxygenase-2 (COX-2), with β-actin as the internal control. In addition, immunofluorescence staining of the M2 macrophage marker (CD206) was conducted to evaluate anti-inflammatory polarization, following the same procedure as forward described.

### *In vitro* angiogenesis assessment

In the scratch wound healing assay, HUVECs were seeded in 6-well plates at a density of 2 × 10⁵ cells/well and cultured until 100% confluence. Linear wounds were created using sterile 200 µL pipette tips, followed by gentle PBS washes to remove detached cells. Cells were then incubated in serum-free medium containing ZIF-8, Au NPs@ZIF-8, Ga, or Au NPs@ZIF-8/Ga (25 µg/mL). To ensure that measurements at different time points were obtained from the identical fields, we pre-selected five reference fields along the scratch for each well and recorded by external marks. Phase-contrast images were acquired at 0 and 12 h using an inverted microscope (Olympus IX73, Japan).

For quantification, scratch closure ratio was measured by calculating the scratch area at each time point using ImageJ software (version 1.53c). The area of the scratch region was defined by manual thresholding, and the percentage of closure was calculated as:

$$\text{Scratchclosure ratio}(\%)=(\mathrm{A}_0-\mathrm{A}_{12})/\mathrm{A}_{0}\times100(\%)$$

A_0_ is the initial wound area and A_12_ is the wound area after incubation for 12 h. For each well, five random fields along the scratch line were analyzed, and the mean value was used for statistical comparison.

For the cell migration in Transwell, HUVECs were serum-starved for 6 h prior to the experiment and resuspended at 5 × 10⁴ cells in 200 µL of serum-free medium. Cells were seeded into the upper chambers of Transwell inserts (8 μm pore size, Falcon, USA), and the lower chambers were filled with complete EGM-2 medium containing nanoparticle treatments. After 24 h incubation, non-migrated cells were removed using cotton swabs. Migrated cells were fixed with 4% paraformaldehyde for 30 min and stained with 0.1% crystal violet for 10 min. Quantification was performed by counting the number of migrated cells in five randomly selected fields per insert under a bright-field microscope (Olympus DP74, Japan).

To investigate the tube formation, pre-chilled 24-well plates were coated with 150 µL Matrigel^®^ Matrix (BD Biosciences, #354230) and incubated at 37 °C for 30 min to allow gel solidification. HUVECs (5 × 10⁴ cells/well) were seeded in Matrigel-coated wells containing nanoparticle-supplemented medium. After 12 h incubation, tube-like structures were imaged using an inverted microscope. Quantification was performed using ImageJ (version 1.53c) with the Angiogenesis Analyzer plugin. Specifically, total tube length, number of junctions, and number of meshes were calculated from at least five randomly selected fields per well.

To evaluate angiogenic protein expression, HUVECs were seeded in 6-well plates at 2 × 10⁵ cells/well and treated with ZIF-8, Au NPs@ZIF-8, Ga, or Au NPs@ZIF-8/Ga (25 µg/mL, or 8 µg/mL Ga) for 72 h. Total protein was extracted, and vascular endothelial growth factor A (VEGFA) expression was analyzed by Western blotting using β-actin as the internal loading control.

### *In vitro* osteogenic differentiation

MC3T3-E1 pre-osteoblasts were seeded in 6-well plates at a density of 2 × 10⁵ cells/well and cultured in complete α-MEM overnight. On the following day, the culture medium was replaced with osteogenic induction medium (OIM; α-MEM supplemented with 10% fetal bovine serum, 1% penicillin/streptomycin, 50 µM ascorbic acid, 10 mM β-glycerophosphate and 10 nM dexamethasone) containing ZIF-8, Au NPs@ZIF-8, Ga, or Au NPs@ZIF-8/Ga at equivalent concentrations (25 µg·mL⁻¹ nanoparticles or 8 µg·mL⁻¹ Ga). Thereafter, OIM was refreshed every 3 days for a total induction period of 21 days. To maintain sustained exposure to particulate treatments, fresh nanoparticles at the same concentration were re-supplemented every 7 days during medium replacement. For the Ga group, Ga was soluble and therefore freshly added at the nominal concentration at each medium change to ensure constant exposure.

On day 14 of induction, cells were fixed with 4% paraformaldehyde for 30 min and stained using a BCIP/NBT ALP detection kit (Beyotime, China) according to the manufacturer’s instructions. The stained plates were imaged under bright-field microscopy to qualitatively assess early osteogenic activity based on ALP expression. After 21 days of induction, extracellular matrix mineralization was assessed by staining with 2% Alizarin Red S (pH 4.2) for 15 min at room temperature. Unbound dye was removed by PBS washes. To quantify calcium deposition, the bound stain was extracted using 10% cetylpyridinium chloride, and absorbance was measured at 562 nm using a microplate reader (Epoch 2, BioTek, USA).

To assess early-stage osteogenic differentiation, immunofluorescence staining for runt-related transcription factor 2 (RUNX2) was performed on day 3. Cells were fixed in 4% paraformaldehyde, permeabilized with 0.1% Triton X-100, blocked with 5% BSA, and incubated with anti-RUNX2 primary antibody (1:200) followed by Alexa Fluor^®^ 488-conjugated secondary antibody. Nuclei were counterstained with Hoechst 33,342 and imaged using confocal microscopy. On day 21, total protein was harvested to assess late-stage markers. The expression of RUNX2 and osteocalcin (OCN) was analyzed by Western blotting using β-actin as the loading control.

### *In vivo* bone regeneration assessment

A critical-sized (5 mm diameter) bilateral calvarial defect model was established in 7-week-old male Sprague–Dawley rats to evaluate the in vivo osteogenic performance of nanoparticle-loaded gelatin methacrylate (GelMA) hydrogels (Fig. S2). All procedures were approved by the Institutional Animal Care and Use Committee of Sun Yat-sen University (Approval No. SYSU-IACUC-2024-001838). All animals were housed under specific pathogen-free (SPF) conditions with a 12/12-hour light/dark cycle, controlled temperature (22 ± 2 °C), and relative humidity (50 ± 10%). In accordance with previously reported bilateral calvarial defect models, each defect site was treated as an independent sample for analysis [38–40]. This approach has been reported in several studies using similar small-animal models.

A total of 45 rats were randomized into five groups (*n* = 9/group): (1) GelMA control, (2) ZIF-8 + GelMA, (3) Au NPs@ZIF-8 + GelMA, (4) Ga + GelMA, and (5) Au NPs@ZIF-8/Ga + GelMA. General anesthesia was induced with a combination of Zoletil (20 mg/kg, i.p.) and xylazine hydrochloride (10 µL, i.m.). Under aseptic conditions, bilateral 5-mm critical-sized calvarial defects were created with a trephine drill under continuous saline irrigation to prevent thermal injury. Each defect was filled with a GelMA hydrogel (10% w/v, EFL-GM-60) containing the assigned nanoparticles or gallic acid. Hydrogels were crosslinked using 365 nm UV light (5 mW/cm², 60 s). Body temperature was maintained using a heating pad during surgery. Postoperative management included subcutaneous analgesia (Antondine, 0.05 mg/kg) and a 3-day course of antibiotics (penicillin, 80,000 IU/day, i.m.). At 2, 4, and 8 weeks post-implantation, rats (*n* = 3 per group per time point) were euthanized, and calvarial samples were harvested and fixed in 4% paraformaldehyde. All animals survived until the predetermined endpoints of the study, with no mortality or unexpected adverse events occurring during the surgical procedure or the postoperative period. Each defect site was analyzed as an independent sample.

Major organs (heart, liver, spleen, lung, and kidney) were harvested at 8 weeks for hematoxylin and eosin (H&E) staining. Calvarial samples of were scanned using a micro-CT system (SkyScan 1276, Bruker, Belgium) under 70 kV and 114 µA, with an isotropic resolution of 10 μm. Three-dimensional reconstructions and bone morphometric analysis were performed using SCANCO Medical software. A standardized cylindrical volume of interest (VOI), centered over the original defect region, was analyzed to determine bone volume to tissue volume ratio (BV/TV), trabecular number (Tb.N), trabecular thickness (Tb.Th), and trabecular separation (Tb.Sp).

Decalcified calvarial specimens (15% EDTA, pH 7.2, 4 weeks) were paraffin-embedded and sectioned (4 μm). H&E staining was used to assess new bone formation and tissue organization. Masson’s trichrome staining was performed to evaluate collagen deposition. Observations were qualitative; no quantitative image analysis was performed.

To evaluate inflammation and neovascularization, immunofluorescence (IF) staining was performed on paraffin sections collected at 2 and 4 weeks. At 2 weeks, iNOS expression was detected using rabbit anti-iNOS (1:200) followed by Alexa Fluor^®^ 488-conjugated secondary antibody. At 4 weeks, dual IF staining was performed for platelet endothelial cell adhesion molecule-1 (CD31/PECAM-1, green, 1:200) and alpha-smooth muscular actin (α-SMA, red, 1:200) to evaluate vascular maturation. VEGFA expression was assessed using species-specific antibodies with Alexa Fluor^®^ 488. At 8 weeks, OCN and RUNX2 expression were assessed via immunohistochemical (IHC) staining. Sections were incubated with rabbit anti-OCN (1:200) and rabbit anti-RUNX2 (1:100), followed by HRP-conjugated secondary antibodies and DAB development. Slides were counterstained with hematoxylin and scanned using a digital slide scanner (Leica Biosystems, AT2).

Detailed protocols for decalcification, HE staining, Masson staining, IF staining and IHC staining are provided in the Supplementary information. All primary antibodies used for Western blotting, immunofluorescence, and immunohistochemistry are summarized in Table S1.

### Statistical analysis

All quantitative data are presented as mean ± standard deviation (SD). Statistical comparisons among multiple groups were performed using one-way analysis of variance (ANOVA) followed by Tukey’s post hoc test. A *p*-value of less than 0.05 was considered statistically significant. All analyses were conducted using GraphPad Prism 8.0 (GraphPad Software, USA).

## Results and discussion

### Synthesis and characterization of Au NPs@ZIF-8/Ga

**Fig. 1:**
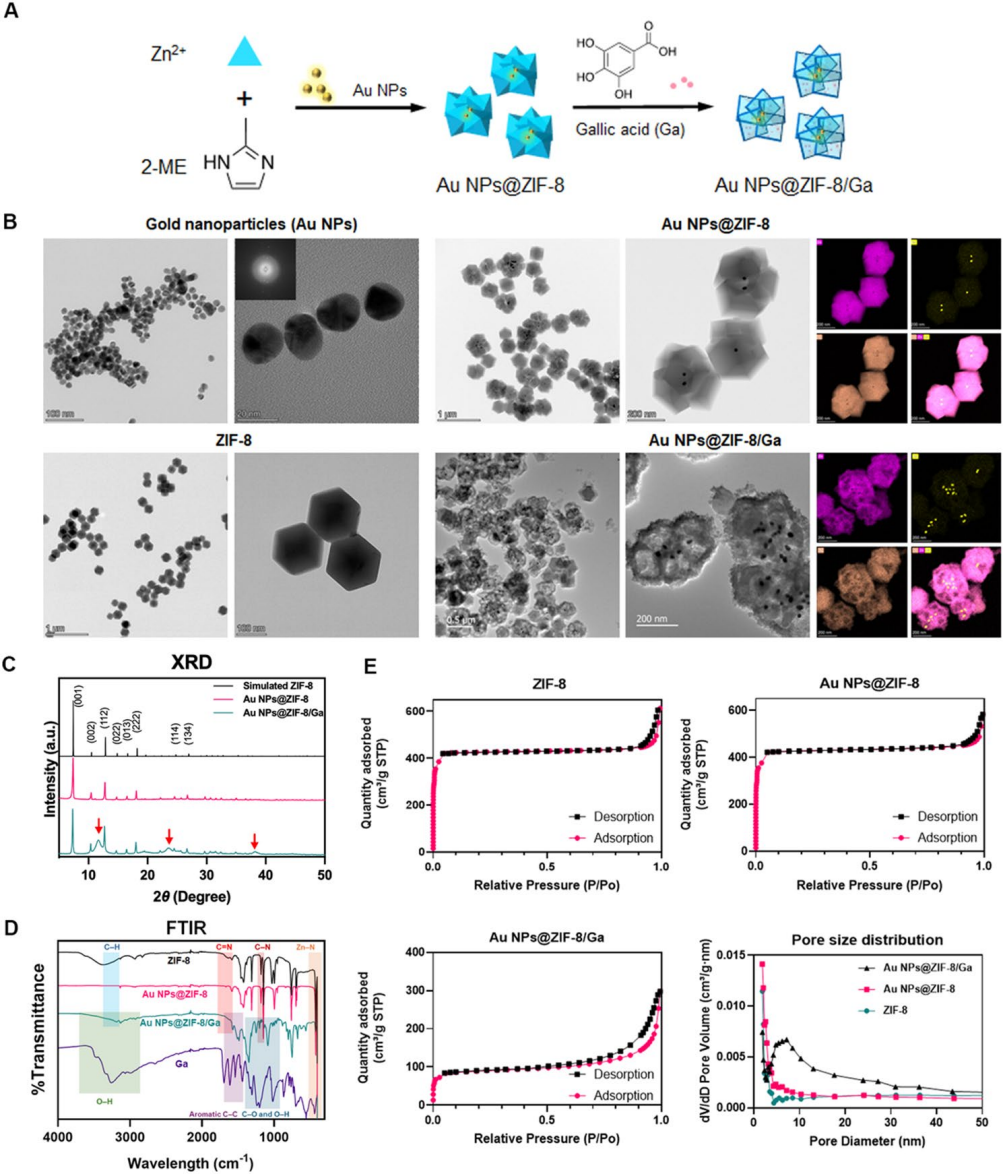
Synthesis and characterization of ZIF-8, Au NPs@ZIF-8, and Au NPs@ZIF-8/Ga. (**A**) Schematic illustration of the synthesis of gold nanoparticles (Au NPs), zeolitic imidazolate framework-8 (ZIF-8), core-shell Au NPs@ZIF-8, and gallic acid-loaded nanoparticles (Au NPs@ZIF-8/Ga.) (**B**) Representative TEM images of Au NPs (top left insert indicates the diffraction pattern), ZIF-8, Au NPs@ZIF-8, and Au NPs@ZIF-8/Ga. Elemental mapping for Au (yellow), Zn (purple), and N (orange) is shown for Au NPs@ZIF-8 and Au NPs@ZIF-8/Ga. (**C**) XRD patterns of Au NPs@ZIF-8 and Au NPs@ZIF-8/Ga, compared to the simulated pattern of pristine ZIF-8 as reference. (**D**) FTIR spectra of ZIF-8, Au NPs@ZIF-8, gallic acid (Ga), and Au NPs@ZIF-8/Ga, highlighting characteristic vibrations from both the framework and the loaded molecules. (**E**) Nitrogen adsorption-desorption isotherms and the corresponding pore size distribution for ZIF-8, Au NPs@ZIF-8, and Au NPs@ZIF-8/Ga

As illustrated in Fig. 1A, the as-synthesized Au NPs were mixed with the precursor solution containing zinc ion and 2-methylimidazole to form Au NPs@ZIF-8 nanocomposites. Subsequently, Ga was loaded to obtain Au NPs@ZIF-8/Ga. TEM revealed the morphological characteristics of nanoparticles across different formulations (Fig. 1B). The synthesized Au NPs appeared uniformly spherical with well-defined boundaries, homogeneous size distribution and diffraction pattern [41]. ZIF-8 particles exhibited a typical rhombic dodecahedral geometry, featuring smooth surfaces and regular contours, indicative of their well-defined crystalline structure [42]. In the Au NPs@ZIF-8 samples, Au NPs were embedded as inner cores within multiple ZIF-8 matrices, forming a classical core–shell nano-flower configuration. After Ga loading, the resulting Au NPs@ZIF-8/Ga preserved the overall core–shell morphology but displayed roughened surfaces and hollow interior regions, suggesting that Ga incorporation induced partial etching and structural modification of the ZIF-8 framework. The wall thickness of more than 30 individual Au NPs@ZIF-8/Ga nanoparticles was measured, yielding an average value of 39.0 ± 9.0 nm. This variation reflects the intrinsic heterogeneity in the extent of hollowing among the nanoparticle population. EDS analysis further confirmed the elemental distribution. Both Au NPs@ZIF-8 and Au NPs@ZIF-8/Ga exhibited a consistent element pattern: gold (Au) signals were concentrated in the core region, whereas the shell contained zinc (Zn), nitrogen (N), and carbon (C), consistent with the chemical composition of ZIF-8 (Zn(C_4_H_5_N_2_)_2_). XRD patterns are shown in Fig. 1C. The characteristic peaks of ZIF-8 were preserved in both ZIF-8 and Au NPs@ZIF-8, indicating that the integration of Au NPs did not disrupt the crystalline integrity of the ZIF-8 shell. Notably, the XRD profile of Au NPs@ZIF-8/Ga retained the key diffraction peaks of ZIF-8, but with the appearance of two broad signals around 11–13° and 23–24° (indicated by red arrows in Fig. 1C), implying that Ga loading introduced partial disorder or amorphization, which is consistent with the hollowed morphology observed via TEM.

FTIR spectroscopy results are displayed in Fig. 1D. ZIF-8 exhibited characteristic absorption bands at 3140 cm⁻¹ (C–H stretching of imidazole ring), 1579 cm⁻¹ (C = N stretching), 1180 cm⁻¹ (C–N stretching), and 420 cm⁻¹ (Zn–N stretching), all in agreement with its expected framework structure. In the Au NPs@ZIF-8 spectrum, these peaks remained sharp and unchanged, indicating that the embedded Au NPs did not alter the surface chemistry of the ZIF-8 shell. No significant Au-related absorption was observed due to the metallic nature of Au NPs in the mid-IR region. Upon Ga loading, additional bands appeared at 3350 cm⁻¹ (O–H stretching of phenolic groups), 1610 cm⁻¹ (C = C stretching in aromatic rings), 1380 cm⁻¹ (C–O–H bending), and 1020 cm⁻¹ (C–O stretching), corresponding to gallic acid functional groups. These bands were retained in Au NPs@ZIF-8/Ga, along with the characteristic peaks of ZIF-8 at 1579, 1180, and 420 cm⁻¹, indicating the structural preservation of the ZIF-8 framework. Notably, the O–H stretching band shifted from 3350 cm⁻¹ (free Ga) to 3250 cm⁻¹ in Au NPs@ZIF-8/Ga, suggesting potential hydrogen bonding or coordination between phenolic hydroxyl groups of Ga and Zn²⁺ sites of ZIF-8. The persistent peak at 1610 cm⁻¹ further confirmed successful Ga incorporation.

Nitrogen adsorption–desorption isotherms of ZIF-8 and Au NPs@ZIF-8 displayed typical type-I profiles (Fig. 1E), with rapid saturation at low relative pressure (P/P₀ < 0.1), indicating microporous structures (< 2 nm). In contrast, Au NPs@ZIF-8/Ga showed a type-H3 hysteresis loop at P/P₀ = 0.5–1.0 and a distinct mesoporous peak in the pore size distribution curve (5–10 nm), implying that Ga loading induced framework etching and mesopore formation. BET surface area analysis revealed that Au NPs@ZIF-8/Ga exhibited a significantly reduced surface area (352.39 m²/g) compared to both ZIF-8 (1791.66 m²/g) and Au NPs@ZIF-8 (1783.91 m²/g), highlighting the structural impact of Ga incorporation. This decrease was mainly attributed to structural etching induced by Ga loading, which led to pore enlargement and partial occupation of micropores by gallic acid. The loading efficiency of gallic acid in Au NPs@ZIF-8/Ga was calculated to be 32.04%, as detailed in the Supplementary Information (Fig. S1 and Table S2).

The release profiles of both Ga and Zn²⁺ from the Au NPs@ZIF-8/Ga composite exhibited a similar two-phase pattern, characterized by an initial burst within the first 7 h followed by a sustained release up to 240 h (Figs. S3, S4). This synchronized kinetics suggests a shared release mechanism governed by the diffusion of the drug/ion from and degradation of the ZIF-8 framework. The initial burst is attributable to the rapid desorption of surface-bound drug molecules/ions, while the subsequent prolonged release reflects the gradual diffusion of GA and ions from the etched interior network.

### Biocompatibility assessment of Au NPs@ZIF-8/Ga

**Fig. 2:**
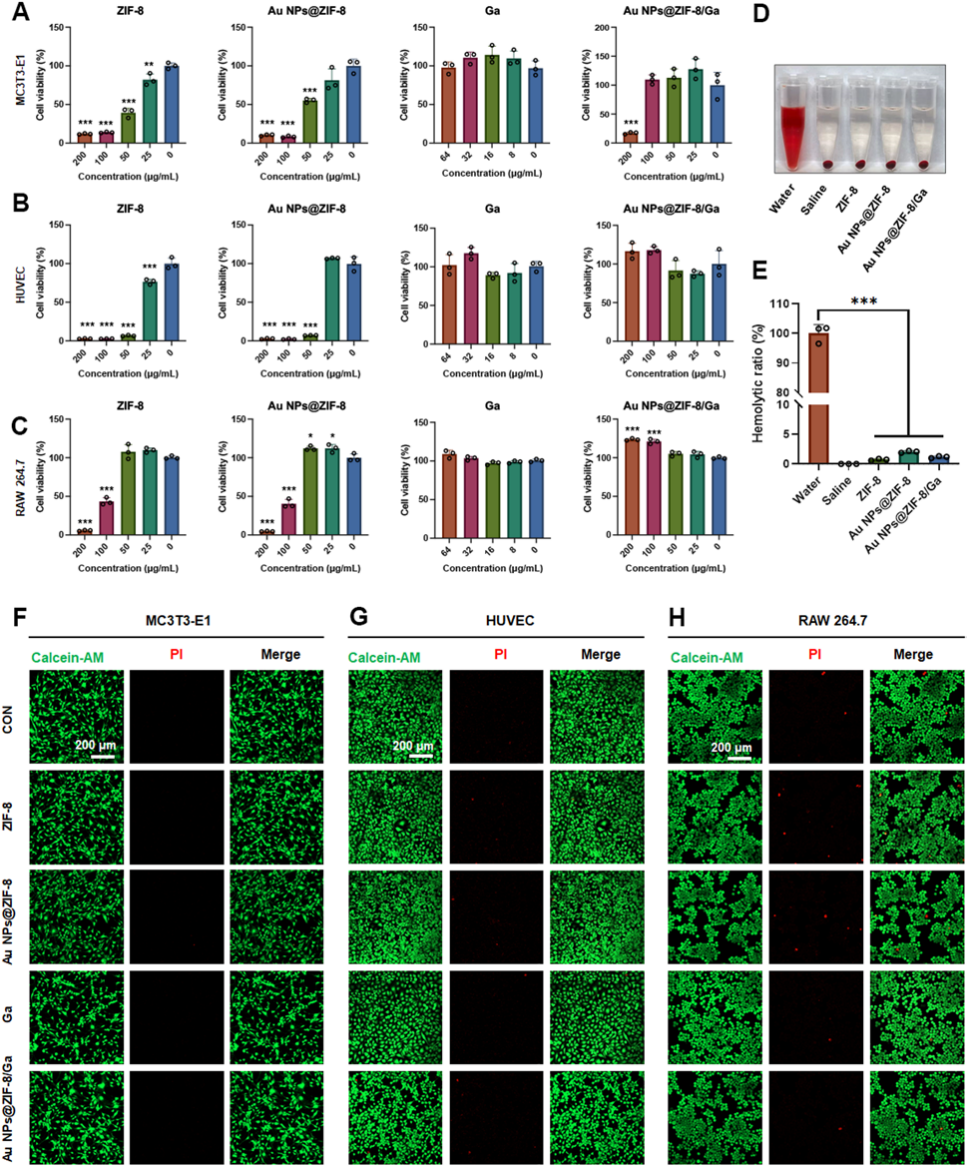
Biocompatibility evaluation of Au NPs@ZIF-8/Ga. (**A**–**C**) Cell viability of MC3T3-E1 preosteoblasts, HUVECs, and RAW 264.7 macrophages after 48 h treatment with different nanoparticles and gallic acid (Ga). (**D**, **E**) Hemolytic activity and quantitative analysis of hemolysis rates (%) after incubation of nanoparticles with red blood cells for 3 h. (**F**–**H**) Representative live/dead fluorescence staining of MC3T3-E1 cells, HUVECs, and RAW 264.7 macrophages. Live cells were stained with Calcein-AM (green), and dead cells with propidium iodide (PI, red). All data are presented as mean ± SD (*n* = 3 independent experiments). Statistical significance was determined as *p < 0.05, **p < 0.01, ***p < 0.001 vs. control group

The cytocompatibility of ZIF-8, Au NPs@ZIF-8, Au NPs@ZIF-8/Ga (25, 50, 100, and 200 µg/mL), and free gallic acid (8, 16, 32, and 64 µg/mL) was evaluated using a CCK-8 assay in MC3T3-E1 preosteoblasts, HUVECs, and RAW 264.7 macrophages after incubation for 24 h (Fig. S5) and 48 h (Fig. 2A–C). Untreated cells cultured in standard medium served as the negative control group. ZIF-8 (25, 50, 100, and 200 µg/mL) and Au NPs@ZIF-8 (50, 100, and 200 µg/mL) significantly reduced the viability of MC3T3-E1 and HUVECs (*p < 0.05), while RAW 264.7 cells exhibited higher tolerance, showing significant cytotoxicity only at 100 and 200 µg/mL for both ZIF-8 and Au NPs@ZIF-8. In contrast, free Ga exhibited no detectable cytotoxic effects across the tested concentration range (8–64 µg/mL) in the three cell lines. Importantly, Au NPs@ZIF-8/Ga exhibited substantially improved biocompatibility compared to ZIF-8 and Au NPs@ZIF-8. For MC3T3-E1 cells, reduced viability was observed only at 200 µg/mL. In HUVECs and RAW 264.7 cells, no significant cytotoxicity was detected even at the highest tested dose of 200 µg/mL. The hemocompatibility and overall biosafety of the materials were further confirmed. Hemolysis assays showed hemolysis rates below 2% for both ZIF-8 and Au NPs@ZIF-8/Ga at 25 µg/mL (Fig. 2D, E), well below the ISO 10993-4 hemocompatibility threshold [43]. Live/dead staining of MC3T3-E1, HUVECs, and RAW 264.7 cells further validated this result, showing comparable fluorescence between nanoparticle-treated and control groups at 25 µg/mL (Fig. 2F–H). To further evaluate long-term biocompatibility, CCK-8 assays were extended to 7 days in all three cell types (Fig. S6). After 7 days of treatment with nanoparticles (25 µg/mL) and gallic acid (Ga, 8 µg/mL), MC3T3-E1 and RAW 264.7 cells exhibited no significant change in viability. Interestingly, HUVECs displayed a significant increase in cell viability in the Ga and Au NPs@ZIF-8/Ga groups compared with the control, indicating a positive effect on endothelial cell proliferation. Taken together, these results confirm the minimal cytotoxicity of ZIF-8, Au NPs@ZIF-8, and Au NPs@ZIF-8/Ga over extended culture periods, and support the selection of 25 µg/mL as the working concentration for subsequent biological evaluations, corresponding to ~ 8 µg/mL Ga in the Au NPs@ZIF-8/Ga group based on its loading efficiency.

According to the Ga release profile of Au NPs@ZIF-8/Ga, although the actual concentrations of Ga released from Au NPs@ZIF-8/Ga were much lower than the concentrations of free Ga (8–64 µg/mL) used in the in-vitro assays. The free Ga groups were originally designed based on the total loading amount of Ga in the nanoparticles, serving as a reference to illustrate the maximum potential bioactivity of Ga. The lower released concentration, in contrast, reflects the sustained-release characteristics of the nanocomposite, which is a key design feature for potential therapeutic applications where gradual delivery is advantageous.

According to the results, ZIF-8 and Au NPs@ZIF-8 exhibited similar cytotoxicity profiles, suggesting that Au NPs incorporation or nanoflower construction did not considerably alter the intrinsic toxicity of the ZIF-8 framework. In contrast, Au NPs@ZIF-8/Ga showed significantly reduced cytotoxicity, highlighting the protective effect of Ga loading and its potential to enhance the biocompatibility of ZIF-8-based platforms for biomedical use.

Based on these findings, we propose several possible mechanisms underlying the reduced cytotoxicity of Au NPs@ZIF-8/Ga. Firstly, Ga incorporation may modulate nanoparticle surface properties and induce the formation of hollow nanoflower structures, which differ from the typical octahedral structure of ZIF-8. The structural changes may potentially influence biocompatibility and contribute to the reduced cytotoxicity, but further experimental validation is needed to confirm this hypothesis. Secondly, as excessive reactive oxygen species (ROS) contribute to the cytotoxicity of ZIF-8 [44], the antioxidant activity of Ga, particularly through its mitochondrial ROS-scavenging activity, may counteract this effect and has additionally been reported to inhibit apoptosis by suppressing the caspase-3 pathway [45]. Collectively, the loading of Ga in the nanoflowers could significantly enhance the biocompatibility while the related molecular pathways involved remain to be fully elucidated.

### Au NPs@ZIF-8/Ga attenuate inflammation by inhibiting the NF-κB signaling pathway

To further investigate the anti-inflammatory effect of Au NPs@ZIF-8/Ga, an in vitro inflammation model was established using RAW 264.7 macrophages stimulated with LPS to mimic acute inflammatory conditions following bone augmentation. Then we assessed the activation of the NF-κB signaling pathway, a classic regulator of inflammation by studying the key markers: the phosphorylation and nuclear translocation of the p65 subunit.

**Fig. 3:**
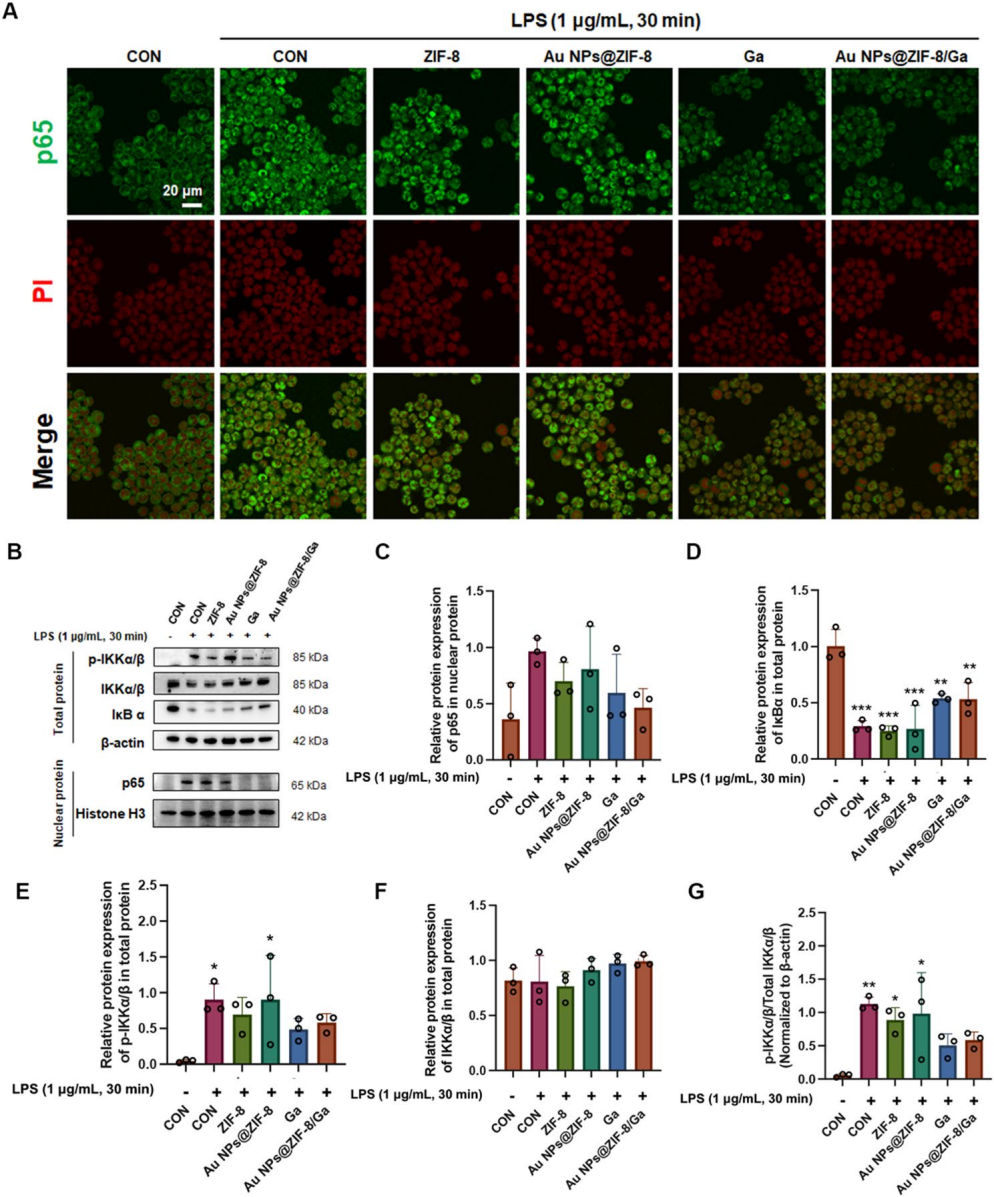
Au NPs@ZIF-8/Ga modulate inflammatory responses by suppressing NF-κB signaling pathway in vitro. (**A**) Immunofluorescence staining of NF-κB p65 nuclear translocation in RAW 264.7 macrophages (green: p65; red: nuclei stained with propidium iodide, PI). (**B**) Western blot and (**C**–**F**) corresponding densitometric quantification analysis of key proteins in NF-κB pathway, including p65 in nuclear extracts (normalized to Histone H3), IκBα, phosphorylated IKKα/β (p-IKKα/β), and total IKKα/β in total protein (normalized to β-actin). (**G**) Quantification of IKKα/β phosphorylation levels, expressed as the ratio of p-IKKα/β to total IKKα/β. Data are presented as mean ± SD (*n* = 3 independent experiments). *p < 0.05, **p < 0.01, ***p < 0.001 vs. negative control group

Immunofluorescence staining at 30 min post-LPS stimulation revealed marked nuclear translocation of the p65 subunit in the LPS-treated group (positive control), ZIF-8, and Au NPs@ZIF-8 groups, indicating activation of the NF-κB signaling pathway (Fig. 3A). In contrast, Ga and Au NPs@ZIF-8/Ga treatments significantly attenuated the nuclear accumulation of p65 (^###^*p* < 0.001 vs. LPS-positive control) (Fig. S7). These observations were corroborated by western blotting of nuclear protein extracts (Fig. 3B, C), which revealed a downward trend in nuclear p65 levels after treatment. Au NPs@ZIF-8/Ga showed the most notable reduction compared with the LPS control, despite the absence of statistically significant differences. Mechanistically, NF-κB is usually retained in the cytoplasm under basal conditions through binding to its inhibitor IκBα. Upon stimulation with LPS (1 µg/mL), the upstream kinase IKKα/β is phosphorylated to form p-IKKα/β, which subsequently phosphorylates IκBα. The phosphorylated IκBα undergoes ubiquitin-mediated degradation via the proteasome pathway, releasing p65, which then translocates into the nucleus to initiate NF-κB-dependent gene transcription. This process is characterized by a decrease in IκBα expression and an increase in nuclear p65.

Western blot analysis showed that the phosphorylation level of IKKα/β (expressed as the p-IKKα/β to total IKKα/β ratio) exhibited a downward trend in the Ga and Au NPs@ZIF-8/Ga groups compared with the LPS control, although the differences did not reach statistical significance (Fig. 3G).The expression of IκBα was higher in Ga and Au NPs@ZIF-8/Ga groups compared to the LPS group, though not fully restored to the level of the negative control, suggesting that these treatments partially protected IκBα from degradation (Fig. 3D). Collectively, these data suggest that Au NPs@ZIF-8/Ga exert potent anti-inflammatory effects by inhibiting phosphorylation of IKKα/β, preventing IκBα degradation, and suppressing nuclear translocation of p65, thereby blocking downstream NF-κB–mediated gene transcription.

**Fig. 4:**
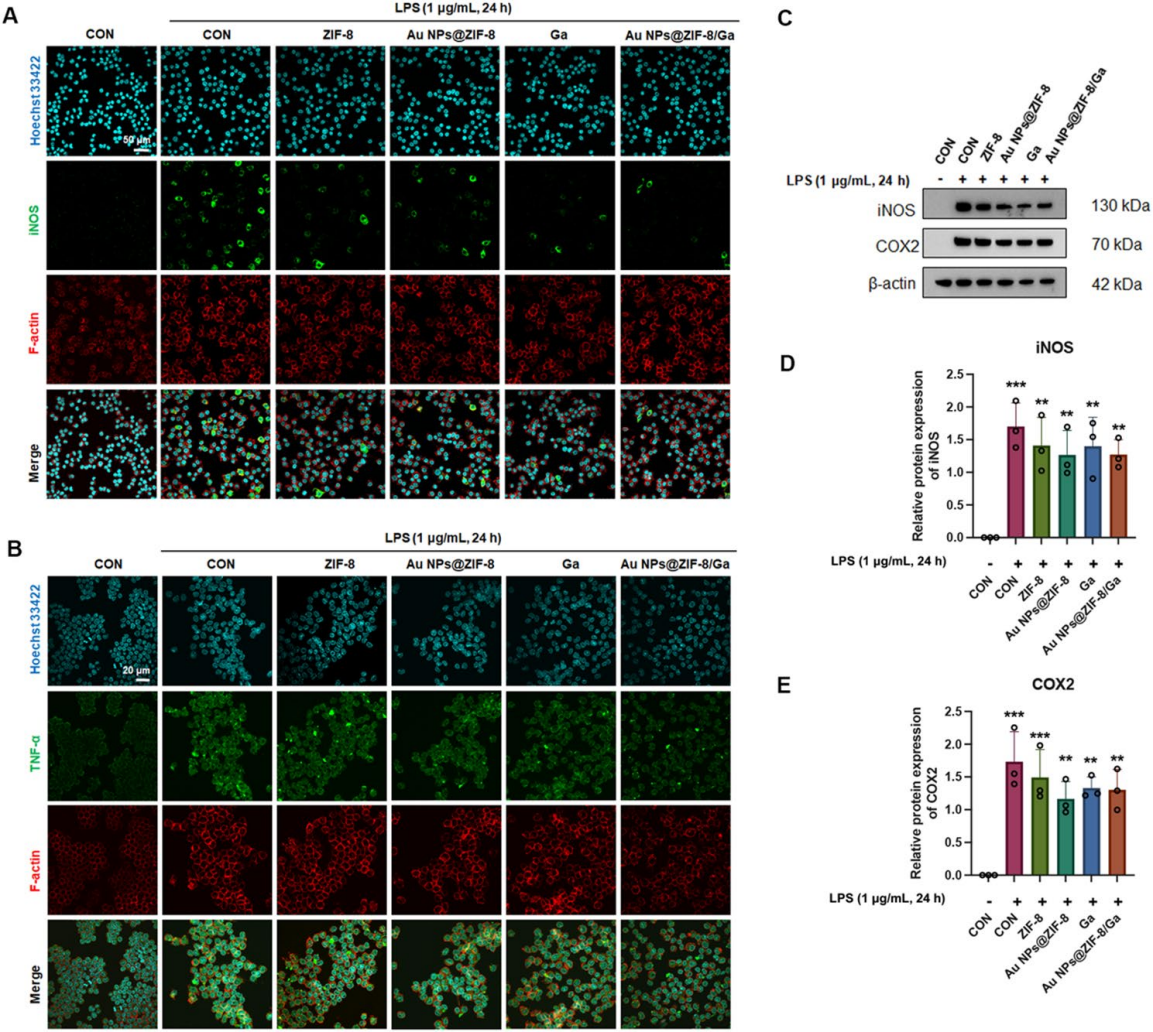
Au NPs@ZIF-8/Ga modulate inflammatory responses via NF-κB signaling in vitro. (**A**) Immunofluorescence staining of iNOS (green), phalloidin (red), and nuclei (Hoechst 33342, blue). (**B**) Immunofluorescence staining of TNF-α (green), phalloidin (red), and nuclei (Hoechst 33342, blue). (**C**) Western blot analysis and (**D**, **E**) densitometric quantification of iNOS and COX-2 expression, normalized to β-actin. Data are presented as mean ± SD (*n* = 3 independent experiments). **p < 0.01, ***p < 0.001 vs. negative control group

In addition to the suppression of p65 nuclear translocation and upstream signaling events, downstream inflammatory mediators were analyzed to further validate the anti-inflammatory effects of Au NPs@ZIF-8/Ga. Specifically, the expression levels of iNOS and TNF-α were assessed in RAW 264.7 macrophages after 24 h of LPS stimulation. Immunofluorescence staining and the average fluorescence intensity revealed the markedly elevated expression of both markers in all LPS-treated groups (Fig. 4A, B and Fig. S8), whereas treatment with Ga or Au NPs@ZIF-8/Ga significantly attenuated their expression. from immunofluorescence (IF) images. These findings were further supported by Western blot analysis, which showed a clear downward trend in the expression of iNOS and COX-2 in both the Ga- and Au NPs@ZIF-8/Ga-treated groups compared with the LPS control, although the differences did not reach statistical significance (Fig. 4C–E). These results provide evidence that Ga incorporation confers potent anti-inflammatory activity through suppression of NF-κΒ-dependent proinflammatory mediator production. These findings support the dual therapeutic action of Au NPs@ZIF-8/Ga: (i) inhibiting NF-κB activation through suppression of p65 phosphorylation and nuclear translocation, and (ii) reducing the expression of proinflammatory mediators. Through simultaneous attenuation of inflammation and preservation of osteoblast function, Au NPs@ZIF-8/Ga contribute to the establishment of a regenerative microenvironment conducive to effective bone repair.

To further examine whether nanoparticles also effect the resolution phase of inflammation, the M2 macrophage marker CD206 was evaluated by immunofluorescence staining. ZIF-8, Au NPs@ZIF-8, Ga and Au NPs@ZIF-8/Ga exhibited enhanced CD206 expression compared with the LPS-positive control group, indicating promoted macrophage polarization toward the M2 phenotype (Fig. S9). However, no significant difference was observed among the four treated groups, suggesting comparable M2-inducing capability.

In the context of therapeutic bone regeneration, local inflammatory responses at the defect site frequently hinder tissue repair. This challenge is particularly evident in alveolar bone augmentation, where surgical trauma, incomplete wound closure, and exposure to the oral microbiota often exacerbate early postoperative inflammation [46]. Uncontrolled inflammation has been shown to impair osteogenesis and delay bone healing, highlighting the need for timely anti-inflammatory interventions to support regenerative outcomes. The NF-κB signaling pathway plays a key role in the regulation of inflammation and has been implicated in negatively modulating bone regeneration [47–49]. It exerts these effects by promoting β-catenin degradation and suppressing the osteogenic differentiation of mesenchymal stem cells (MSCs) [46, 48]. Additionally, NF-κB activation downregulates key osteogenic genes such as collagen I (COL-1) and OCN, thereby impairing extracellular matrix formation by osteoblasts [50]. In contrast, targeted inhibition of the IKK/NF-κB axis has shown the effect to enhance bone mass and volume in transgenic models, and specific disruption of the p65–Smad4 interaction has been reported to restore bone morphogenetic protein (BMP)-induced osteogenesis without compromising inflammation resolution [47, 51, 52]. Furthermore, NF-κB downstream effectors such as TNF-α, iNOS, and COX-2 play pivotal roles in acute inflammatory responses and have been widely associated with osteogenesis suppression through osteoclast activation and disruption of bone remodeling [53–55]. For instance, TNF-α overexpression delays fracture healing, whereas pharmacological inhibition of TNF-α has been shown to improve bone regeneration [55]. In our study, Au NPs@ZIF-8/Ga downregulated TNF-α, iNOS, and COX-2 expression in LPS-stimulated macrophages, suggesting their potential to mitigate acute inflammation and support early bone healing.

Nevertheless, we acknowledge that our in vitro design primarily reflects preventive effects, as materials were applied before LPS stimulation. This does not fully represent therapeutic efficacy in pre-established inflammatory states, which warrants further validation in future studies.

### Au NPs@ZIF-8/Ga promoting angiogenesis *in vitro*

**Fig. 5:**
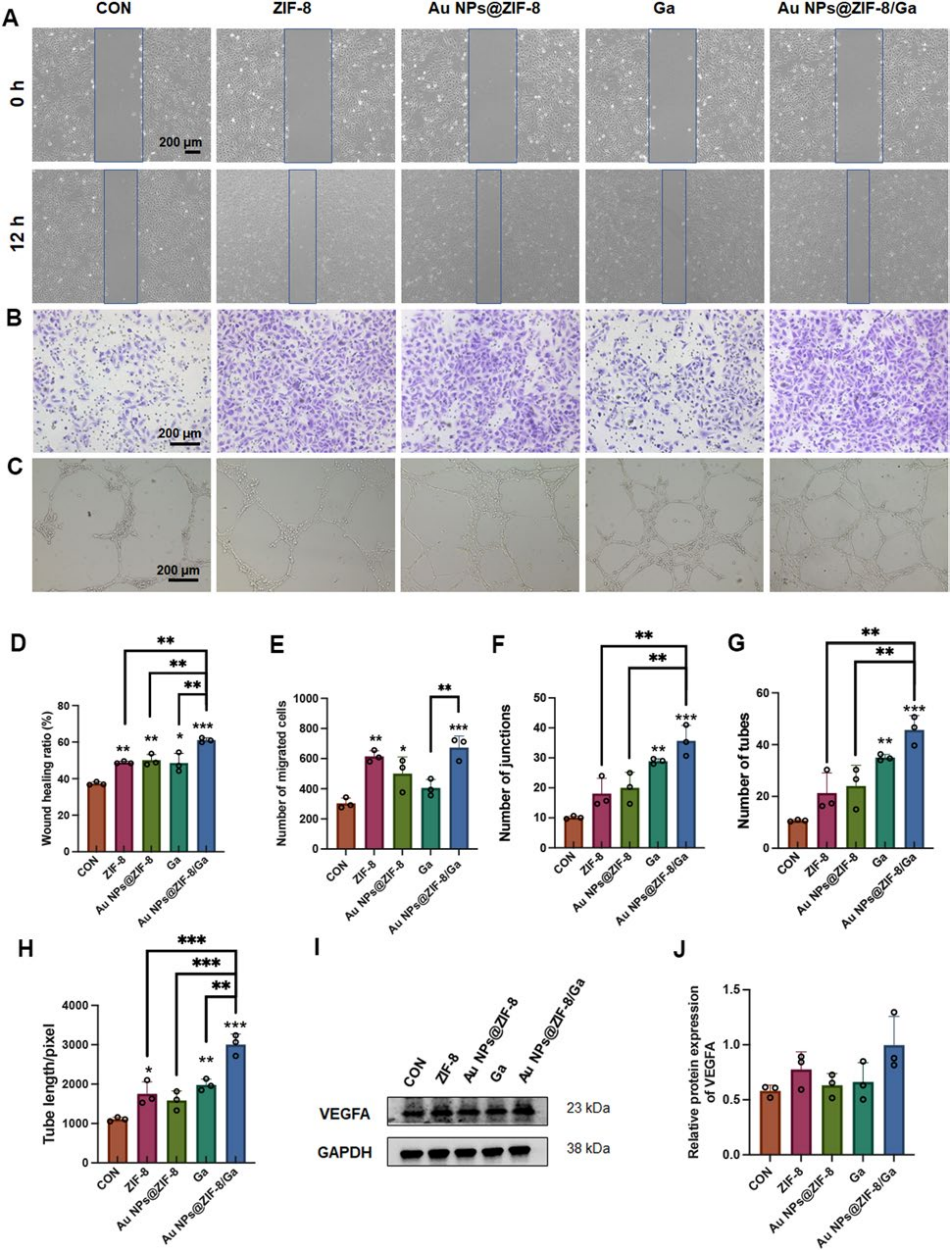
Angiogenic potential of HUVECs modulated by Au NPs@ZIF-8/Ga. (**A**) Representative images of scratch wound healing assay at 0 h and 12 h. Dashed blue boxes indicate the initial wound boundary. (**B**) Images of Transwell migration assay showing HUVECs in the lower chamber after 24 h of culture. (**C**) Representative tube formation images of HUVECs seeded on Matrigel. (**D**) Quantitative analysis of scratch closure rates. (**E**) Quantification of migrated cells in the Transwell assay. (**F**–**H**) Quantification of total tube length, number of branch points, and number of tubular structures in the tube formation assay. (**I**) Western blot analysis of VEGF-A expression in HUVECs, with GAPDH as a loading control. (**J**) Densitometric quantification of VEGF-A expression based on Western blot results. All data are presented as mean ± SD (*n* = 3 independent experiments). Asterisks directly above individual bars indicate significant differences compared to the control group. Lines connecting two bars with asterisks denote significant differences between those specific groups (*p < 0.05, **p < 0.01, ***p < 0.001)

To assess the angiogenic potential of Au NPs@ZIF-8/Ga, we first evaluated the migration capacity of HUVECs. Scratch assay results and quantitative analysis (Fig. 5A, D) demonstrated significantly enhanced scratch closure ratio in the ZIF-8, Au NPs@ZIF-8, Ga, and Au NPs@ZIF-8/Ga groups compared to the control (*p < 0.05), indicating improved endothelial migration. Although the ZIF-8 group did not show statistically significant changes, a trend toward increased migration was observed. Among all treatments, Au NPs@ZIF-8/Ga exhibited the most pronounced pro-migratory effect, outperforming ZIF-8, Au NPs@ZIF-8, and Ga individually (**p < 0.01). Meanwhile, in the Transwell migration assay, HUVECs were seeded in the upper chamber containing serum-free medium, while the lower chamber contained complete medium supplemented with the respective treatments. After 24 h, ZIF-8, Au NPs@ZIF-8, and Au NPs@ZIF-8/Ga significantly increased the number of migrated cells compared to the control (*p < 0.05), whereas free Ga exhibited no notable chemotactic effect. Notably, Au NPs@ZIF-8/Ga induced comparable or slightly higher levels of chemotaxis-induced migration than ZIF-8 and Au NPs@ZIF-8 (Fig. 5B, E), suggesting that all ZIF-8-based nanocomposites intrinsically promote endothelial recruitment. Regarding the tube formation assay, Au NPs@ZIF-8/Ga significantly improved in vitro capillary network formation in Matrigel^®^, as evidenced by increased total tube length, number of junctions, and number of tube-like structures (Fig. 5C, F–H, ***p < 0.001). Given its well-reported role in vascular regulation, VEGF serves as a key angiogenic factor that activates downstream PI3K/Akt [56] and MAPK pathways [57] via binding to VEGF receptors, thereby promoting endothelial cell proliferation, migration, and vascular permeability. Moreover, VEGF also functions as a crucial coupling factor between angiogenesis and osteogenesis [58], indirectly enhancing osteogenic differentiation by activating RUNX2 and Osterix expression in osteoblasts [59, 60]. Western blot analysis indicated an increasing trend in VEGFA expression in the Au NPs@ZIF-8/Ga group, although the difference was not statistically significant. Together with the enhanced cell migration and tube formation observed in HUVEC assays, these findings suggest that Au NPs@ZIF-8/Ga promote endothelial activation and angiogenic responses.

Previous studies have shown that ZIF-8 promotes angiogenesis by inducing VEGF expression in bone marrow mesenchymal stem cells (BMSCs) [45]. In our study, a direct pro-angiogenic effect on HUVECs was observed, as evidenced by enhanced VEGFA expression and tube formation. Such effects are likely influenced by Zn²⁺ release, which has been reported to activate the HIF-1α/VEGF axis and promote angiogenesis [61]. Additionally, zinc-stimulated VEGF-A secretion by HUVECs can activate the PI3K/Akt pathway in BMSCs, thereby forming a positive feedback loop that coordinates angiogenesis and osteogenesis [62]. Notably, Au NPs@ZIF-8/Ga outperformed ZIF-8 alone in promoting angiogenic behaviors. This enhancement may be attributed to the antioxidative effects of sustained Ga release [63, 64], which may help maintain endothelial function under oxidative stress. Although Ga has previously been reported to exert anti-angiogenic effects in cancer-related contexts [65–67], our study found that Ga treatment at 8 µg/mL improved HUVEC migration, tube formation, and VEGFA expression compared to controls. These discrepancies may stem from differences in concentration (lower in our study than the 20 µg/mL commonly used in anti-metastasis models) and cell type specificity. Further investigations are warranted to elucidate the underlying mechanisms. In tissue-engineered bone constructs, integrating angiogenic function is essential for successful graft integration and survival. Vascularization has been widely recognized as a precondition for effective bone regeneration [59, 68]. By enhancing HUVECs migration, promoting tube formation, Au NPs@ZIF-8/Ga demonstrate strong angiogenic potential, offering a promising platform for vascularized bone regeneration. These materials may serve as an effective strategy for large-scale bone defect repair and other regenerative applications that require rapid neovascularization.

### Au NPs@ZIF-8/Ga promoting osteogenic differentiation *in vitro*

**Fig. 6:**
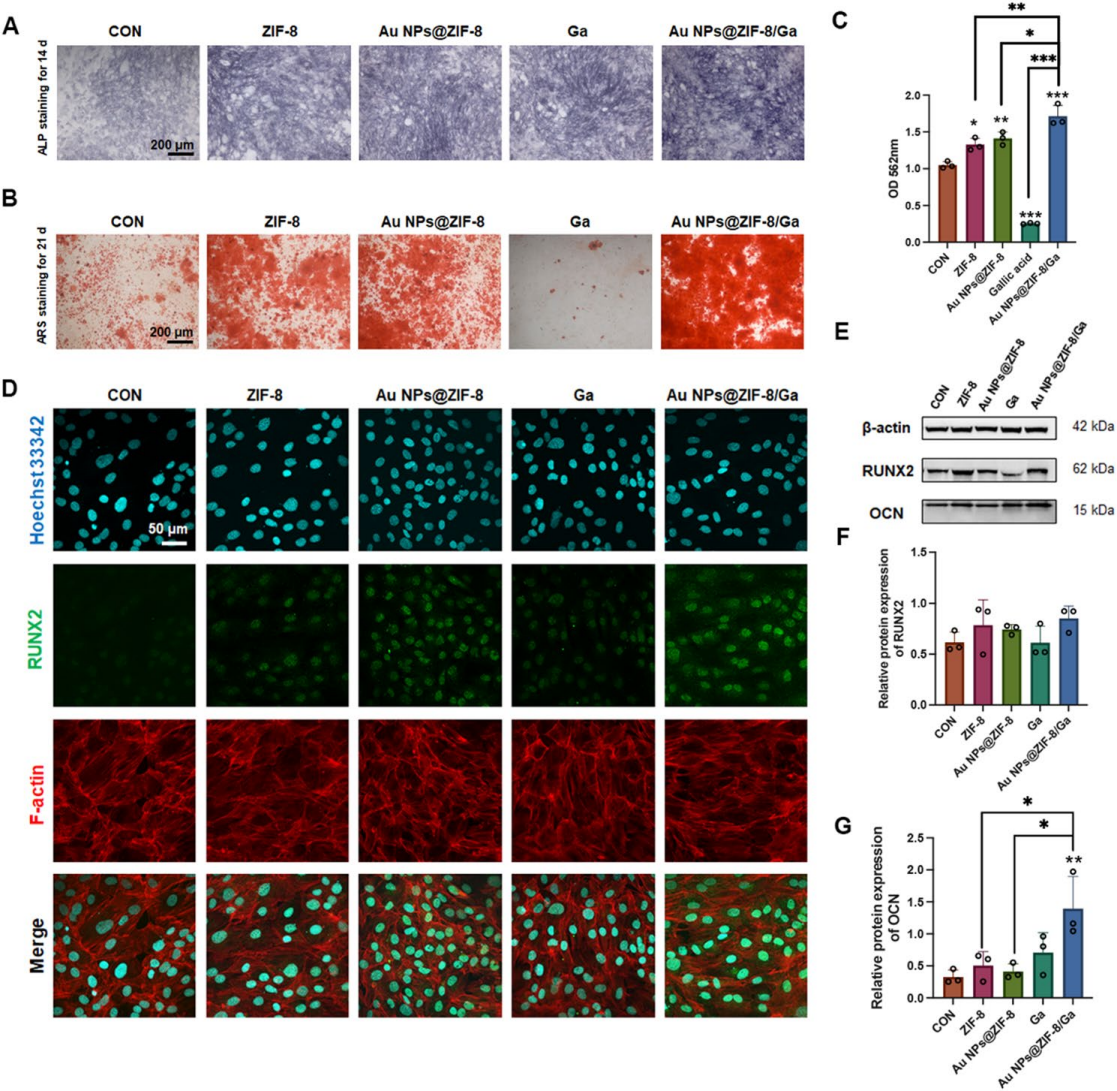
Osteogenic differentiation of MC3T3-E1 cells enhanced by Au NPs@ZIF-8/Ga.(**A**) ALP staining of MC3T3-E1 cells after 14 days of osteogenic induction. (**B**) Alizarin Red S staining and (**C**) quantification of mineralized nodules (red) after 21 days of induction. (**D**) Immunofluorescence staining of RUNX2 (green) in MC3T3-E1 cells after 3 days of induction. (**E**–**G**) Western blot analysis of RUNX2 and OCN protein expression, and corresponding densitometric quantification after 21 days of induction. All data are presented as mean ± SD (*n* = 3 independent experiments). Asterisks directly above individual bars indicate significant differences compared to the control group. Lines connecting two bars with asterisks denote significant differences between those specific groups (*p < 0.05, **p < 0.01, ***p < 0.001)

To evaluate the osteogenic effects of Au NPs@ZIF-8/Ga, we conducted a series of in vitro assays using MC3T3-E1 cells under osteoinductive conditions. ALP staining on day 14 revealed increased ALP activity in all experimental groups compared to the control, with Au NPs@ZIF-8/Ga inducing the most pronounced enhancement (Fig. 6A). Alizarin Red S (ARS) staining and corresponding quantitative analysis on day 21 further demonstrated significant mineralized nodule formation in the ZIF-8, Au NPs@ZIF-8, and Au NPs@ZIF-8/Ga groups (Fig. 6B, C), while free Ga showed minimal mineralization, likely due to long-term accumulation and chelating property disrupting the process of mineralization and calcium deposition. Moreover, the expression of osteogenic markers was examined to verify differentiation outcomes. Immunofluorescence staining of RUNX2 (Fig. 6D and Fig. S10) on day 3 and Western blot analysis of RUNX2 and OCN on day 21 confirmed the upregulation of osteogenic proteins, particularly in the Au NPs@ZIF-8/Ga group, which exhibited the highest expression levels among all treatments (Fig. 6E–G).

The enhanced osteogenesis observed in the Au NPs@ZIF-8/Ga group is likely due to the sustained release of gallic acid (Ga) and zinc ions (Zn²⁺). Ga has been shown to promote osteogenic differentiation via the GPR35/GSK3β/β-catenin signaling axis and to counteract cadmium-induced suppression of osteogenesis by activating ALP and promoting collagen synthesis [30, 69]. In addition, Ga-containing materials have been increasingly utilized to functionalize implants and enhance hydrogel-based bone regeneration [70, 71]. ZIF-8 also plays an essential role in promoting osteogenesis. Gradual release of Zn²⁺ could enhance ALP activity and matrix mineralization via MAPK pathway activation [72] and support biomineralization by facilitating hydroxyapatite nucleation [73–75]. Moreover, nanosized ZIF-8 particles can be internalized by osteoblasts and MSCs, leading to intracellular Zn²⁺ release within acidic lysosomal compartments. This, in turn, triggers receptor-mediated signaling cascades such as MAPK/ERK, promotes RUNX2 expression, and accelerates osteogenic commitment. Although Au NPs have been reported to support osteogenesis through Wnt/β-catenin signaling and autophagy activation [76–79], Au NPs@ZIF-8 did not exhibit superior osteoinductive capacity compared to ZIF-8 alone in our study. This may be due to the Au NPs being encapsulated as cores within the composite and thus not directly interacting with the cells.

In summary, the synergistic effect of sustained Ga and Zn²⁺ release from Au NPs@ZIF-8/Ga enhanced osteogenic differentiation and mineral deposition. These findings underscore the material’s potential as an effective osteoinductive platform in bone regeneration applications.

### Au NPs@ZIF-8/Ga enhance bone regeneration in a rat cranial defect model

**Fig. 7:**
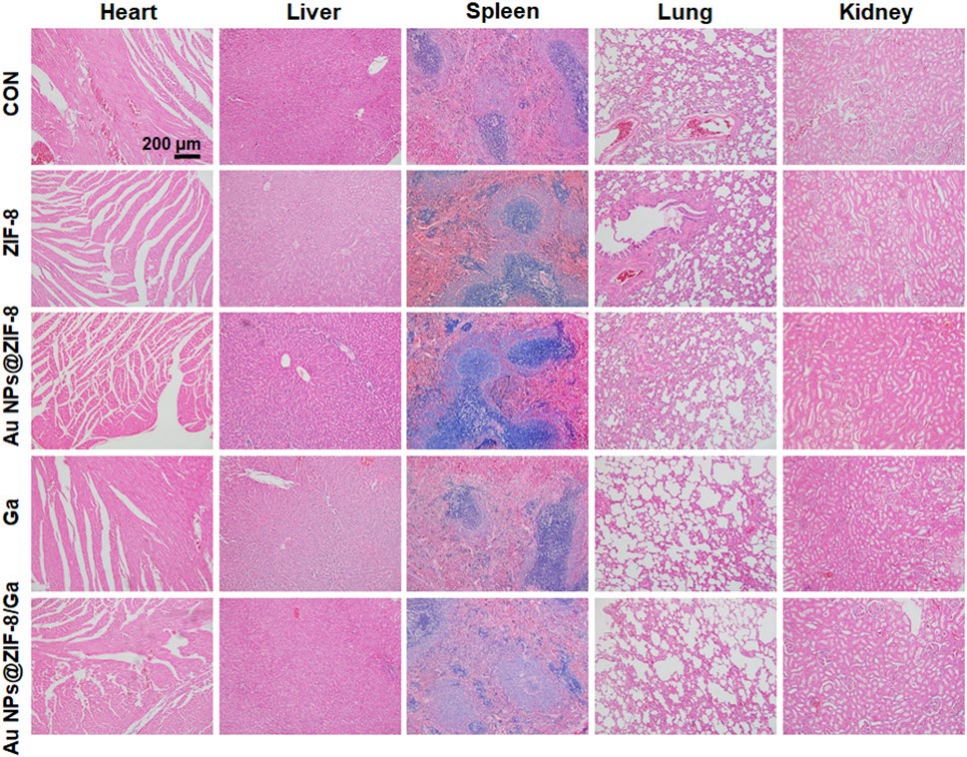
Histological evaluation of major organs following scaffold implantation. Representative H&E staining images of heart, liver, spleen, lung, and kidney harvested from SD rats at 8 weeks post-implantation

To investigate the in vivo effects of the nanoparticles, they were embedded in GelMA-based scaffolds [80], followed by implantation in the cranial defects of SD rats, thereby enabling the sustained release of the nanoparticles and maintaining their activity at the bone defect site. The in vivo biocompatibility of the scaffolds was first evaluated by H&E staining of major organs. No histopathological abnormalities were observed in the heart, liver, spleen, lung, or kidney across all treatment groups after 8 weeks of scaffold implantation, confirming the systemic safety of the nanomaterials (Fig. 7).

**Fig. 8:**
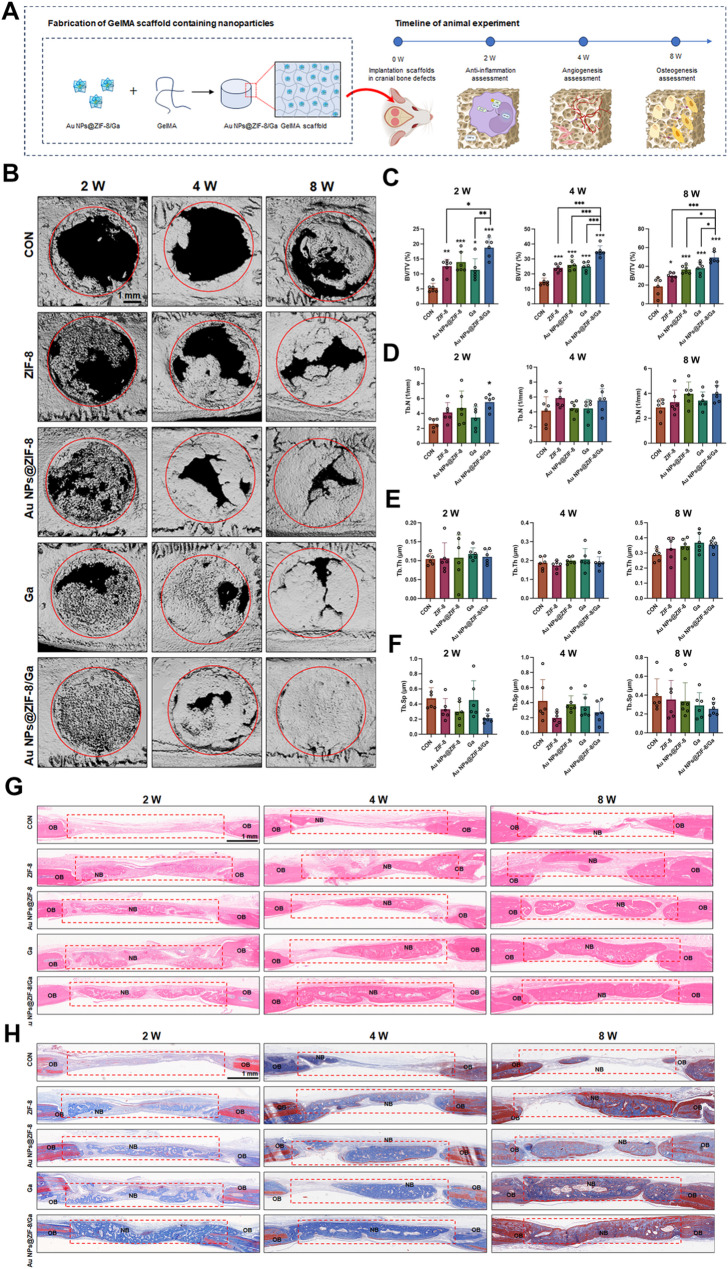
Enhanced cranial bone regeneration mediated by Au NPs@ZIF-8/Ga in a rat critical-sized defect model. (**A**) Schematic illustration of the experimental timeline and surgical procedure. Au NPs@ZIF-8/Ga were encapsulated in GelMA hydrogel prior to implantation. (**B**) Representative micro-CT reconstructions of the cranial defect region (highlighted by red circles) at 2, 4, and 8 weeks post-surgery. (**C**–**F**) Quantitative analysis of BV/TV, Tb.N, Tb.Th, and Tb.Sp at 2, 4, and 8 weeks post-surgery (*n* = 6 defects per group per time point). (**G**) H&E staining of defect regions (outlined by red dashed boxes) at 2, 4, and 8 weeks. NB: newly formed bone; OB: original bone. (**H**) Masson’s trichrome staining of the same regions showing collagen deposition and matrix remodeling at each timepoint. All data are presented as mean ± SD. Asterisks directly above individual bars indicate significant differences compared to the control group. Lines connecting two bars with asterisks denote significant differences between those specific groups (*p < 0.05, **p < 0.01, ***p < 0.001)

The in vivo experimental timeline is outlined in Fig. 8A. Micro-CT) revealed progressive new bone formation in all experimental groups at 2, 4, and 8 weeks, with the Au NPs@ZIF-8/Ga group consistently exhibiting the most robust bone regeneration (Fig. 8B). At 2 weeks, early bone deposition partially filled the defect area, while in the Au NPs@ZIF-8/Ga group, low-density new bone had nearly bridged the entire defect. By 8 weeks, highly mineralized bone closely resembling native architecture was observed, particularly in the Au NPs@ZIF-8/Ga group, where the defect appeared almost completely regenerated. Quantitative micro-CT analysis further supported these findings. BV/TV was significantly increased in all treatment groups versus controls at all time points (Fig. 8C). Au NPs@ZIF-8/Ga achieved the highest BV/TV values of 18.77 ± 3.27% (2 weeks), 35.04 ± 3.75% (4 weeks), and 49.43 ± 6.15% (8 weeks), confirming its superior osteogenic capacity. Tb.N was significantly elevated in the Au NPs@ZIF-8/Ga groups at 2 weeks (Fig. 8D), suggesting a denser trabecular network. No significant differences were observed in Tb.Th across most groups, except for a modest increase in the Ga group at 8 weeks (Fig. 8E). Au NPs@ZIF-8/Ga showed a decreasing tendency in Tb.Sp compared to control group at 2, 4 and 8 weeks, suggesting the formation of more compact trabecular structures (Fig. 8F).

In-vivo studies showed consistently higher BV/TV values in the Au NPs@ZIF-8 group compared to the ZIF-8 group at all time points. In contrast, in vitro experiments revealed no significant difference in osteogenic capacity. These results suggest that Au NPs may enhance bone regeneration at the in vivo microenvironment.

Histological analysis supports the micro-CT results. At 2 weeks, the control group showed fibrous connective tissue with minimal new bone, while the experimental groups, especially Au NPs@ZIF-8/Ga, displayed discontinuous trabeculae embedded within cellular matrices. By 4 weeks, the Au NPs@ZIF-8/Ga group exhibited thicker trabeculae and darker eosinophilic staining, indicating active osteoid deposition. At 8 weeks, H&E staining revealed dense, interconnected trabeculae fully spanning the defect in the Au NPs@ZIF-8/Ga group (Fig. 8G). Masson’s trichrome staining further confirmed these findings, showing abundant collagen deposition and increasing maturity of bone tissue from 2 to 8 weeks, with Au NPs@ZIF-8/Ga demonstrating the most pronounced effect (Fig. 8H).

Together, imaging and histological evidence across all time points illustrate the dynamic progression of bone repair from early fibrous tissue replacement to mature mineralized bone. Au NPs@ZIF-8/Ga-loaded GelMA scaffolds accelerated this process, improving both the rate and quality of bone regeneration. These results highlight the synergistic therapeutic potential of Ga and ZIF-8 in enhancing scaffold-mediated bone repair.

**Fig. 9:**
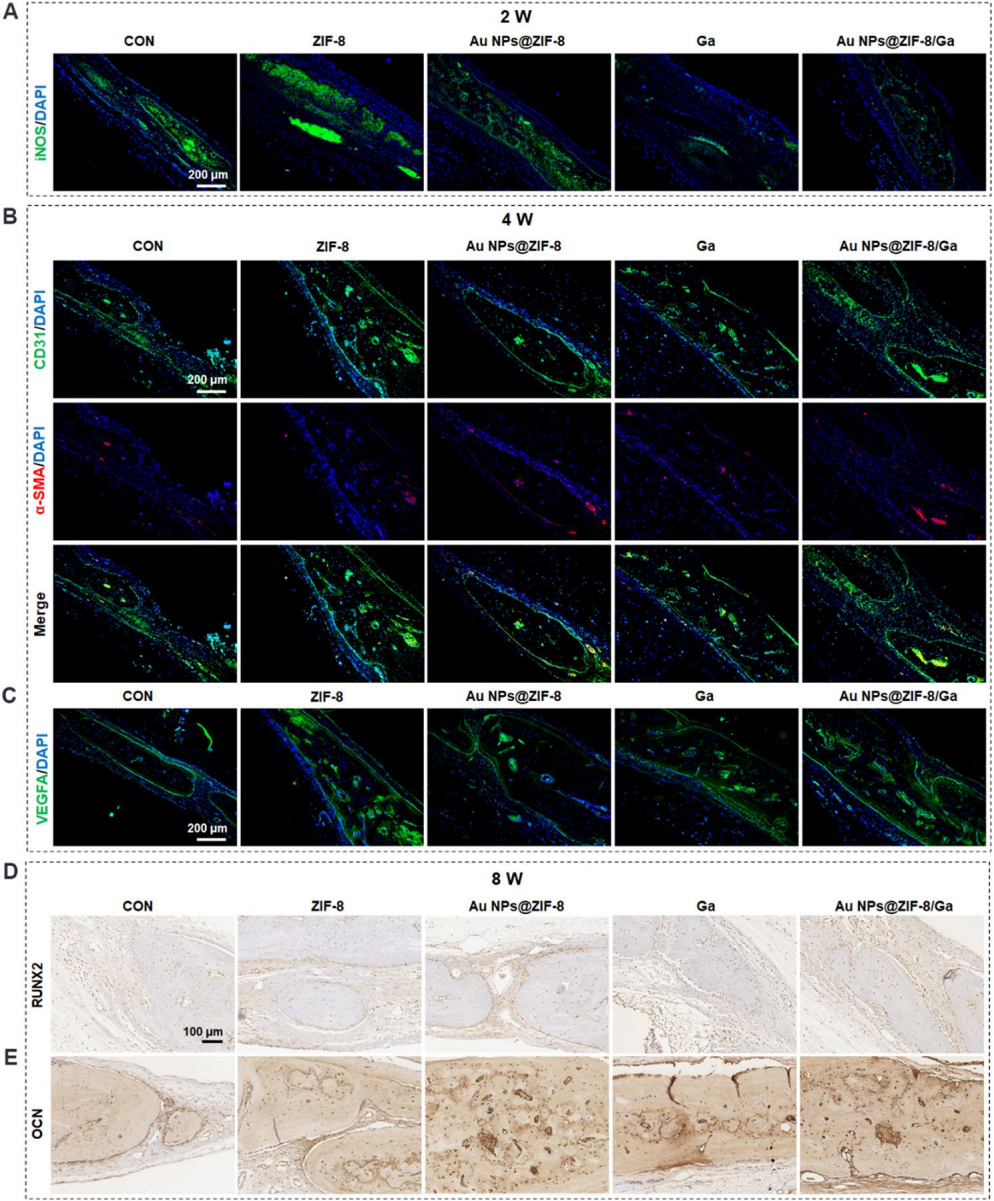
Modulatory effects of Au NPs@ZIF-8/Ga on inflammation, angiogenesis, and osteogenesis during bone regeneration in vivo. (**A**) Immunofluorescence staining of iNOS (green) and DAPI (blue) in defect regions at 2 weeks post-surgery. (**B**) Dual immunofluorescence staining of platelet endothelial cell adhesion molecule-1 (CD31, green) and alpha-smooth muscular actin (α-SMA, red) with DAPI (blue) in 4-week sections to assess neovascularization and vascular maturation. (**C**) Immunofluorescence staining of VEGFA (green) and DAPI (blue) in 4-week sections. (**D**) Immunohistochemical staining of OCN and (**E**) RUNX2 in 8-week sections to evaluate osteogenic activity

The anti-inflammatory, pro-angiogenic, and pro-osteogenic properties of Au NPs@ZIF-8/Ga were studied in vitro. To further investigate their therapeutic mechanism in vivo, we performed IF and IHC staining of bone defect sections at selected timepoints. At postoperative week 2, IF staining for iNOS was conducted to evaluate local inflammation. As shown in Fig. 9A, iNOS expression was markedly elevated in the ZIF-8 group but downregulated in both the Ga and Au NPs@ZIF-8/Ga groups compared to the control. These findings suggest that ZIF-8 alone may exacerbate inflammatory responses in vivo, while the sustained release of gallic acid from Au NPs@ZIF-8/Ga effectively suppresses iNOS expression, indicating a favorable anti-inflammatory effect. By week 4, immunofluorescence staining for CD31, α-SMA, and VEGFA was used to assess vascularization (Fig. 9B, C). CD31 marks neovascular structures, while α-SMA labels mature smooth muscle cells within stable vessels. Au NPs@ZIF-8/Ga and Au NPs@ZIF-8 increased vessel formation compared to control, particularly in terms of mature vessel density. Additionally, VEGFA expression was highest in the Au NPs@ZIF-8/Ga group, especially within fibrous tissues and new trabeculae, supporting its capacity to enhance angiogenesis during the mid-phase of bone repair.

At week 8, IHC staining was performed to assess osteogenic markers RUNX2 and OCN (Fig. 9D, E). All treatment groups exhibited elevated levels of both proteins compared to control. Interestingly, while in vitro osteogenic potential between ZIF-8 and Au NPs@ZIF-8 appeared comparable, in vivo analysis presented higher OCN and RUNX2 expression in the Au NPs@ZIF-8 group. This finding aligns with the BV/TV outcomes observed in the micro-CT analysis (Fig. 8C). This difference may be due to the longer observation window in vivo, allowing gradual breakdown of the ZIF-8 shell and sustained release of Au NPs, thereby contributing to osteogenesis. Among all groups, Au NPs@ZIF-8/Ga induced the strongest upregulation of RUNX2 and OCN. This may reflect a synergistic effect, where its early anti-inflammatory action and mid-stage pro-angiogenic activity create an optimal environment for subsequent osteogenesis. Notably, while the pro-osteogenic contribution of Au NPs was not prominent in short-term in vitro settings, it became evident in vivo after prolonged exposure. This supports recent findings that Au NPs can modulate macrophage polarization toward the M2 phenotype and promote osteogenic differentiation via immunoregulatory and Wnt/β-catenin signaling pathways [81, 82]. M2 macrophages secrete growth factors such as VEGF and TGF-β, which further enhance neovascularization and osteogenesis.

Taken together, these results highlight the multifaceted therapeutic benefits of Au NPs@ZIF-8/Ga, including its anti-inflammatory, pro-angiogenic, and pro-osteogenic activities. By sequentially modulating the bone healing microenvironment at different stages, beginning with inflammation suppression, followed by vascularization, and ultimately promoting bone formation, this nanocomposite scaffold effectively enhances and accelerates bone regeneration. These findings suggest that Au NPs@ZIF-8/Ga is a promising material for scaffold-assisted bone repair.

From a clinical perspective, this platform shows potential for managing complex bone defects, particularly those associated with chronic inflammation or insufficient vascularization. However, further validation in large animal models and orthotopic defect sites is highly necessary. In addition, long-term degradation behavior, biosafety under physiological conditions, and controlled release kinetics should be thoroughly investigated. Moreover, the relatively small sample size (3 rats per group per timepoint) used in the present study represents a limitation, which was constrained by ethical considerations and adherence to the 3R principles (Replacement, Reduction, Refinement). Future studies will focus on optimizing the composition and structure of the scaffold, clarifying cell-specific mechanisms of action, and developing combination strategies with stem cells or biological agents. These efforts aim to improve translational potential and broaden clinical applicability. Overall, Au NPs@ZIF-8/Ga represents a strong candidate for next-generation bone tissue engineering.

## Conclusion

In conclusion, we successfully fabricated core–shell structured nanoparticles (Au NPs@ZIF-8) by in situ growth of ZIF-8 on gold nanoparticle cores, followed by Ga loading to form Au NPs@ZIF-8/Ga, a hollow nanoflower-like material. While maintaining its core–shell architecture, Ga induced structural etching of ZIF-8, resulting in altered morphology and reduced surface area. Both in vitro and in vivo studies demonstrated that Au NPs@ZIF-8/Ga possesses anti-inflammatory, pro-angiogenic, and pro-osteogenic activities. These findings suggest its potential as a bioactive nanoplatform for enhancing bone regeneration. Further studies are needed to elucidate its underlying mechanisms and explore broader therapeutic applications.

## Supplementary Information

Supplementary Material 1

## Data Availability

No datasets were generated or analysed during the current study.

## Abbreviations

ALP: Alkaline phosphatase
Au NPs: Gold nanoparticles
BET: Brunauer–Emmett–Teller
BMP: Bone morphogenetic protein
BMSCs: Bone marrow mesenchymal stem cells
BV/TV: Bone volume to tissue volume ratio
CD31/PECAM-1: Platelet endothelial cell adhesion molecule-1
COL-1: Collagen I
COX-2: Cyclooxygenase-2
EDS: Energy-dispersive spectroscopy
FBS: Fetal bovine serum
FTIR: Fourier-transform infrared spectroscopy
Ga: Gallic acid
GelMA: Gelatin methacrylate
H&E: Hematoxylin and eosin
IF: Immunofluorescence
IHC: Immunohistochemical
iNOS: Inducible nitric oxide synthase
LE: Loading efficiency
LPS: Lipopolysaccharide
Micro-CT: Micro-computed tomography
MOF: Metal-organic framework
MSCs: Mesenchymal stem cells
NF-κB: Nuclear factor kappa B
OCN: Osteocalcin
OIM: Osteogenic induction medium
PI: Propidium iodide
PVP: Polyvinylpyrrolidone
RBCs: Red blood cells
ROS: Reactive oxygen species
RUNX2: Runt-related transcription factor 2
SD: Standard deviation
Tb.N: Trabecular number
Tb.Sp: Trabecular separation
Tb.Th: Trabecular thickness
TEM: Transmission electron microscopy
TNF-α: Tumor necrosis factor-α
VEGFA: Vascular endothelial growth factor A
XRD: X-ray diffraction
ZIF-8: Zeolitic imidazolate framework-8
Zn²⁺: Zinc ions
α-SMA: Alpha-smooth muscular actin
