# Supplementary material for: Bioengineered hollow nanoflowers to synergistically modulate inflammation, angiogenesis and osteogenesis for enhancing repair of bone defects
Source: J Nanobiotechnology. 2025 Dec 5;24:27. doi: 10.1186/s12951-025-03891-0 (PMC12797472; doi:10.1186/s12951-025-03891-0)
Supplement: Supplementary file 1 — Supplementary Material 1 [file 12951_2025_3891_MOESM1_ESM.docx]

**Supporting Information**

**Title: Bioengineered Hollow Nanoflowers to Synergistically Modulate Inflammation, Angiogenesis and Osteogenesis for Enhancing Repair of Bone Defects**

**Experimental Section**

**Gallic Acid Loading Efficiency (LE) of Au NPs@ZIF-8/Ga**

To determine the loading efficiency of gallic acid (Ga) into Au NPs@ZIF-8/Ga nanocomposites, absorbance-based quantification was performed using a UV–vis spectrophotometer at 256 nm (Figure S1). A standard calibration curve was generated, and the Ga concentrations in all relevant solutions were determined based on optical density (OD) values (Table S1).

During the synthesis process, supernatants were collected at three stages:

(1) before Ga addition (initial Ga solution).

(2) after loading (residual unbound Ga).

(3) from washing steps (washed-off Ga).

The amount of Ga successfully incorporated into the nanoparticles was determined by subtracting the amounts detected in the post-loading and wash solutions from the initial input. The loading efficiency (LE, %) was calculated using the following equation:

$$\text{L}\text{oading efficiency of Ga \%= }\frac{W \mathrm{initial}-W residual-W \mathrm{wash}}{\text{W }\text{Au NPs@ZIF-8}\text{/}\text{Ga}}\text{×100\%}$$

W _initial_: total Ga added before loading

W _residual_: unbound Ga remaining in solution after loading

W _wash_: Ga content in washing solution

W _Au NPs@ZIF-8/Ga_: dry weight of the final loaded composite

**Fig. S1.** The standard curve for gallic acid (Ga) solutions was detected at 256 nm by a UV−vis spectrophotometer

**Ga Releasing Study**

To evaluate the release behavior of Ga from Ga@Au NPs@ZIF-8, 5 mg of the sample was dispersed in 1 mL of phosphate-buffered saline (PBS, pH 7.4) in a 1.5 mL microcentrifuge tube. The tubes were incubated at 37 °C under gentle shaking. At predetermined time points (1, 3, 7, 24, 48, 72, 111, 168, and 240 h), the samples were centrifuged at 8000 rpm for 10 min, and 1 mL of the supernatant was collected for analysis. The same volume of fresh PBS (1 mL) was then added to the remaining precipitate to maintain a constant volume for continued release. This procedure was repeated at each sampling point.

The concentration of Ga in the collected supernatants was determined using a UV–Vis spectrophotometer based on the characteristic absorbance of gallic acid, and the cumulative release profile was calculated accordingly.

**Determination of Zn²⁺ releasing**

The release behavior of Zn²⁺ from Au NPs@ZIF-8/Ga was quantified using Inductively Coupled Plasma-Optical Emission Spectrometer (ICP-OES) (Agilent 720ES/OES, USA) in PBS (pH 7.4, 37 °C). At each time points (1, 3, 7, 24, 48, 72, 111, 168, 192, 216 and 240 h), samples were centrifuged at 8000 rpm for 10 min, and supernatants were collected for analysis. The cumulative release was calculated based on calibration curves obtained from Zn²⁺ standards.

**Cell Culture Conditions**

MC3T3-E1 pre-osteoblasts were cultured in α-MEM medium supplemented with 10% fetal bovine serum (FBS) and 1% penicillin-streptomycin (P/S). Cells were maintained at 37°C in a humidified incubator with 5% CO₂ and passaged at 70–80% confluency using 0.25% trypsin-EDTA.

Human umbilical vein endothelial cells (HUVECs) were cultured in Endothelial Cell Growth Medium containing growth supplements. Medium was changed every 2 days and cells at passages 3–6 was used.

RAW 264.7 murine macrophages were maintained in DMEM medium supplemented with 10% FBS and 1% P/S. Cells were cultured under standard incubator conditions and sub-cultured every 2–3 days.

**Protein Extraction and Western Blotting**

Protein Extraction: Cytoplasmic and nuclear proteins were extracted using a nuclear/cytoplasmic protein extraction kit (Beyotime, China) according to the manufacturer’s protocol. Total cellular proteins were extracted using RIPA buffer containing protease and phosphatase inhibitors (Beyotime, China).

Western Blotting: Protein concentrations were determined using a BCA assay kit (Thermo Fisher). Equal amounts of protein (20–30 μg) were separated via SDS-PAGE and transferred onto PVDF membranes. Membranes were blocked with 5% non-fat milk in TBST and incubated with primary antibodies overnight at 4°C. After washing, membranes were incubated with HRP-conjugated secondary antibodies and visualized using enhanced chemiluminescence (ECL) reagents.

**Immunofluorescence (IF) Staining**

Cell-based IF staining: Cells were fixed with 4% paraformaldehyde for 15–30 min and permeabilized with 0.1% Triton X-100. After blocking with 5% BSA for 1 h, samples were incubated with primary antibodies overnight at 4°C. Following PBS washes, Alexa Fluor-conjugated secondary antibodies were added for 1 h at room temperature. Nuclei were counterstained with Hoechst 33342 (10 μg/mL). Fluorescence images were captured using a confocal laser scanning microscope (Olympus, FV3000, Japan).

Paraffin section IF staining: Paraffin-embedded tissue sections were deparaffinized, rehydrated, and subjected to antigen retrieval using citrate buffer (pH 6.0) heated in a microwave. Sections were then blocked with 5% BSA for 1 h and incubated with primary antibodies overnight at 4°C. After PBS washes, Alexa Fluor-conjugated secondary antibodies were applied for 1 h at room temperature. Nuclei were counterstained with DAPI and images were obtained by fluorescence microscope (Olympus, BX63+DP74, Japan).

**Histological Staining Procedures**

Decalcification: Bone samples were decalcified in 15% EDTA (pH 7.2) for 4 weeks at room temperature with constant shaking. EDTA was refreshed every 2–3 days.

Hematoxylin and Eosin (H&E) Staining: Paraffin-embedded sections (4 μm) were deparaffinized, rehydrated, and stained with hematoxylin for 5 min, followed by eosin for 3 min. Slides were dehydrated, cleared in xylene, and mounted.

Masson’s Trichrome Staining: Sections were processed using a commercial Masson staining kit (Solarbio, China) according to the manufacturer’s instructions to assess collagen deposition.

**Immunohistochemistry (IHC)**

Tissue sections were deparaffinized, rehydrated, and subjected to antigen retrieval in citrate buffer (pH 6.0) using microwave heating. Endogenous peroxidase activity was quenched using 3% hydrogen peroxide. Sections were blocked with 5% BSA and incubated with primary antibodies overnight at 4°C. HRP-conjugated secondary antibodies and DAB chromogen were used for detection. Hematoxylin was used for nuclear counterstaining.

**Table S1. Primary antibodies used in this research**

| **Target** | **Host Species** | **Dilution** | **Application** | **Source** |
| --- | --- | --- | --- | --- |
| iNOS | Rabbit | 1:200, 1:1000 | IF, WB | Abmart |
| TNF-α | Rabbit | 1:200 | IF | Affinity |
| CD31 | Rabbit | 1:200 | IF | ZENBIO |
| α-SMA | Mouse | 1:200 | IF | ZENBIO |
| VEGFA | Rabbit | 1:200, 1:1000 | IF, WB | Affinity |
| RUNX2 | Rabbit | 1:100, 1:1000 | IF, WB | CST |
| RUNX2 | Rabbit | 1:100 | IHC | ZENBIO |
| OCN | Rabbit | 1:200, 1:1000 | IHC, WB | Affinity |
| p65 | Rabbit | 1:200, 1:1000 | IF, WB | CST |
| IκBα | Rabbit | 1:1000 | WB | CST |
| p-IKKα/β | Rabbit | 1:1000 | WB | CST |
| IKKα/β | Rabbit | 1:1000 | WB | CST |
| β-actin | Rabbit | 1:2000 | WB | Servicebio |
| Histone H3 | Rabbit | 1:1000 | WB | Abmart |


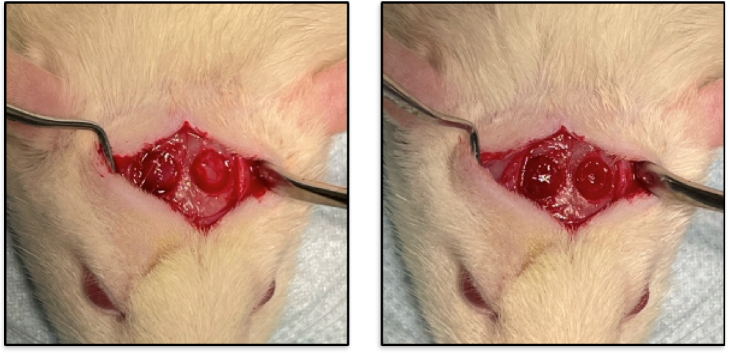


**Fig. S2** Surgical images of cranial bone defect of SD rats.

**Results**

**Gallic Acid Loading Efficiency (LE) of Au NPs@ZIF-8/Ga**

**Table S2.** Correspondence between OD values and concentrations of Ga solution.

|  | Dilution rate | OD_256 nm_ | Concentration (μg/mL) | Volume (mL) | Amount (μg) |
| --- | --- | --- | --- | --- | --- |
| Solution before loading | 100 | 0.897 | 31.59 | 20 | 63181.02 |
| Solution after loading | 100 | 0.172 | 7.014 | 20 | 14028.47 |
| Washing solution | 1 | 2.091 | 72.06 | 20 | 1441.30 |

The calculated loading efficiency of gallic acid in Au NPs@ZIF-8/Ga was 32.04%, indicating that approximately one-third of the initially added Ga was successfully incorporated into the nanocomposites. This result demonstrates effective encapsulation of Ga within the Au NPs@ZIF-8 framework.

**Fig. S3.** Time-dependent accumulate release curve of Ga from Au NPs@ZIF-8/Ga in PBS

**Fig. S4.** Time-dependent accumulate release curve of Zn^2+^ from Au NPs@ZIF-8/Ga in PBS

~~
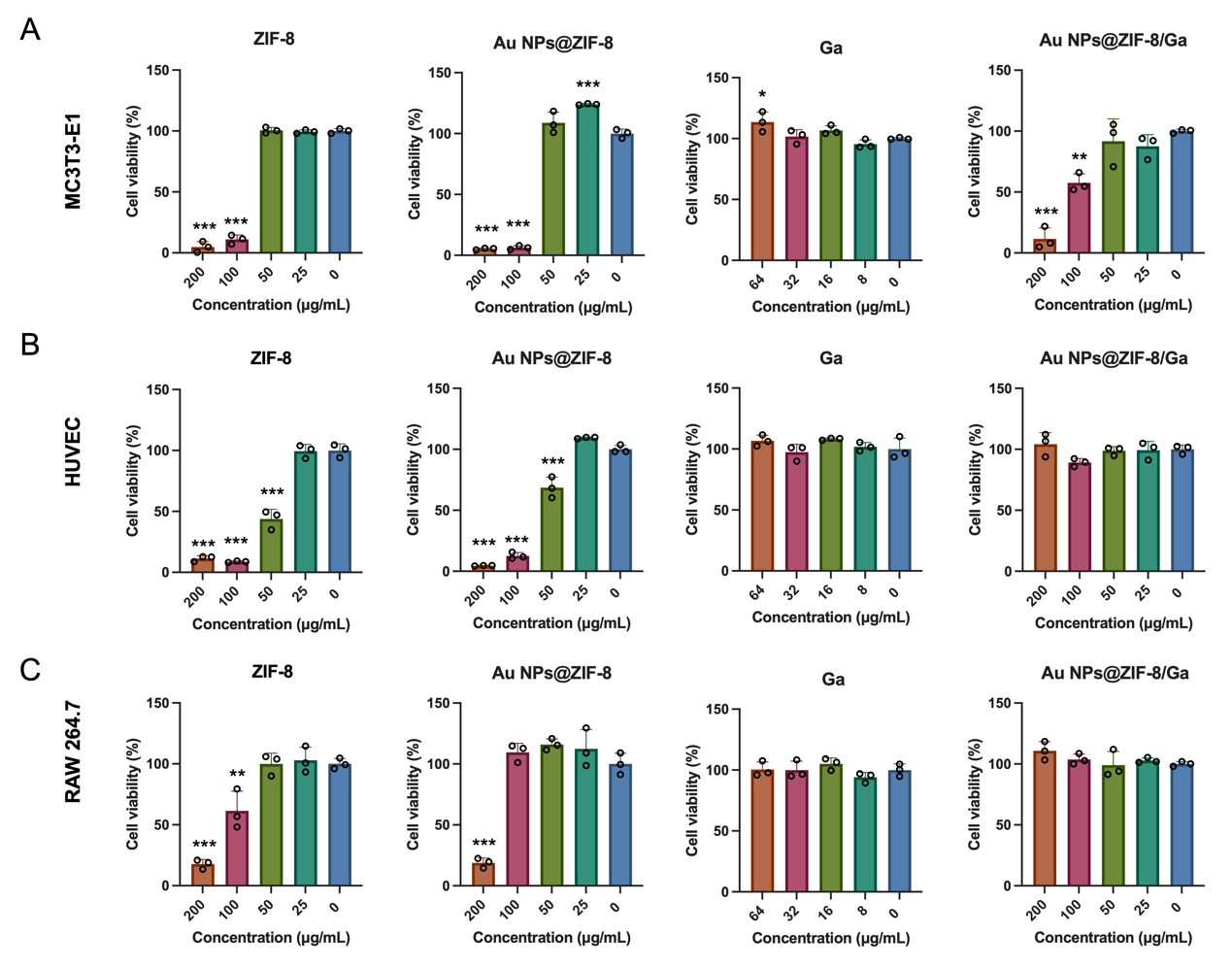
~~

**Fig. S5** Cell viability of MC3T3-E1 (A), HUVECs (B), and RAW 264.7 (C) after 24 h treatment with different nanoparticles (25 μg/mL) and gallic acid (Ga, 8 μg/mL). Statistical significance was determined as **p* < 0.05, ***p* < 0.01, ****p* < 0.001vs. control group.


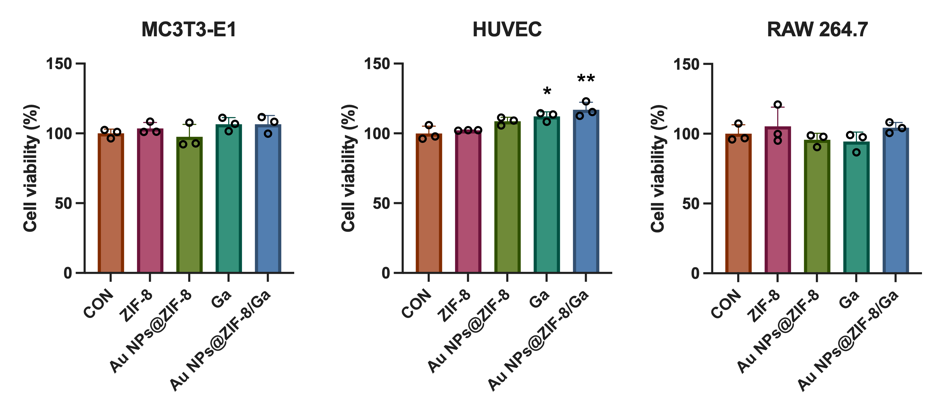


**Fig. S6** Cell viability of MC3T3-E1 preosteoblasts, HUVECs, and RAW 264.7 macrophages after 7 d treatment with different nanoparticles (25 μg/mL) and gallic acid (Ga, 8 μg/mL). Statistical significance was determined as **p* < 0.05, ***p* < 0.01 vs. control group.

**Fig. S7** The nuclear-to-cytoplasmic fluorescence intensity ratio of p65. (^#^means significantly different from positive control group, ^#^*p* < 0.05, ^###^*p* < 0.001; *means significantly different from negative control group, **p* < 0.05, ****p* < 0.05).

**Fig. S8** (A) Quantification of fluorescence intensities of iNOS in RAW 264.7 cells of different groups. (B) Quantification of fluorescence intensities of TNF-α in RAW 264.7 cells of different groups. Fluorescence intensities were calculated under three random fields with image J. N = 3 independent biological replicates, ^###^*p* < 0.001 vs. positive control, and ***p* < 0.001, ****p* < 0.001 vs. negative control.

**Fig. S9.** (A) Immunofluorescence staining of CD206 (green) and nuclei (DAPI, blue) of RAW 264.7. The scale bars = 20 μm. (B) Quantification of fluorescence intensities of CD206 in RAW 264.7 cells of different groups.

**Fig. S10.** Quantification of fluorescence intensities of RUNX2 in MC3T3-E1 of different groups. Fluorescence intensities were calculated under three random fields with image J. N = 3 independent biological replicates, ***p* < 0.01 and ****p* < 0.001.
